# HIV induces synaptic hyperexcitation via cGMP-dependent protein kinase II activation in the FIV infection model

**DOI:** 10.1371/journal.pbio.2005315

**Published:** 2018-07-27

**Authors:** Keira Sztukowski, Kaila Nip, Paige N. Ostwald, Matheus F. Sathler, Julianna L. Sun, Jiayi Shou, Emily T. Jorgensen, Travis E. Brown, John H. Elder, Craig Miller, Franz Hofmann, Sue VandeWoude, Seonil Kim

**Affiliations:** 1 College of Veterinary Medicine and Biomedical Sciences, Colorado State University, Fort Collins, Colorado, United States of America; 2 Cellular and Molecular Biology Graduate Program, Colorado State University, Fort Collins, Colorado, United States of America; 3 Department of Biomedical Sciences, Colorado State University, Fort Collins, Colorado, United States of America; 4 Molecular, Cellular and Integrative Neurosciences, Colorado State University, Fort Collins, Colorado, United States of America; 5 Pharmaceutical Science and Neuroscience, University of Wyoming, Laramie, Wyoming, United States of America; 6 Department of Immunology and Microbiology, The Scripps Research Institute, La Jolla, California, United States of America; 7 Technical University of Munich, Munich, Germany; 8 Department of Microbiology, Immunology, and Pathology, Colorado State University, Fort Collins, Colorado, United States of America; Centre International de Recherche en Infectiologie (CIRI), France

## Abstract

Over half of individuals infected with human immunodeficiency virus (HIV) suffer from HIV-associated neurocognitive disorders (HANDs), yet the molecular mechanisms leading to neuronal dysfunction are poorly understood. Feline immunodeficiency virus (FIV) naturally infects cats and shares its structure, cell tropism, and pathology with HIV, including wide-ranging neurological deficits. We employ FIV as a model to elucidate the molecular pathways underlying HIV-induced neuronal dysfunction, in particular, synaptic alteration. Among HIV-induced neuron-damaging products, HIV envelope glycoprotein gp120 triggers elevation of intracellular Ca^2+^ activity in neurons, stimulating various pathways to damage synaptic functions. We quantify neuronal Ca^2+^ activity using intracellular Ca^2+^ imaging in cultured hippocampal neurons and confirm that FIV envelope glycoprotein gp95 also elevates neuronal Ca^2+^ activity. In addition, we reveal that gp95 interacts with the chemokine receptor, CXCR4, and facilitates the release of intracellular Ca^2+^ by the activation of the endoplasmic reticulum (ER)-associated Ca^2+^ channels, inositol triphosphate receptors (IP3Rs), and synaptic NMDA receptors (NMDARs), similar to HIV gp120. This suggests that HIV gp120 and FIV gp95 share a core pathological process in neurons. Significantly, gp95’s stimulation of NMDARs activates cGMP-dependent protein kinase II (cGKII) through the activation of the neuronal nitric oxide synthase (nNOS)-cGMP pathway, which increases Ca^2+^ release from the ER and promotes surface expression of AMPA receptors, leading to an increase in synaptic activity. Moreover, we culture feline hippocampal neurons and confirm that gp95-induced neuronal Ca^2+^ overactivation is mediated by CXCR4 and cGKII. Finally, cGKII activation is also required for HIV gp120-induced Ca^2+^ hyperactivation. These results thus provide a novel neurobiological mechanism of cGKII-mediated synaptic hyperexcitation in HAND.

## Author summary

Human immunodeficiency virus (HIV)-associated neurocognitive disorders (HANDs) occur in as many as 50% of individuals infected with HIV, including patients receiving combination antiretroviral therapy (cART). Notably, while neuronal death is mitigated with cART, neuronal dysfunction persists. This study investigates HAND-associated alteration of neuronal function, in particular, synaptic activity. The development of therapies designed to prevent HAND requires a detailed understanding of pathogenic mechanisms—processes difficult to study in humans. Here, we develop a feline immunodeficiency virus (FIV) model to study this question. FIV is genetically and functionally similar to HIV and produces a naturally occurring AIDS that is frequently associated with the development of neurological disease. We demonstrate that FIV and HIV share the core cellular pathway that alters neuronal activity via aberrant neuronal activation of cGMP-dependent protein kinase II. Thus, FIV infection of cats can be a valuable model to investigate the neurobiological mechanisms of HAND-associated neuropathogenesis.

## Introduction

Human immunodeficiency virus (HIV)-associated neurocognitive disorders (HANDs) occur in as many as 50% of individuals infected with HIV, including patients receiving combination antiretroviral therapy (cART) [1]. HANDs range from mild neurological disorder (MND) and asymptomatic neurocognitive impairment (ANI) to severe and disabling dementia, and confer an increased risk of early mortality [1,2]. As cART enables individuals infected with HIV to survive to older ages, the prevalence of HAND continues to increase [1], and thus treatments targeting HIV’s pathological processes in the brain are greatly needed. Previous knowledge of HAND neuropathogenesis is dependent on studies that have been predominantly carried out in the pre-ART era [3]. In fact, the majority of basic research on HAND has been focused on evaluating neuronal damage in the context of active viral replication and outcomes related to encephalitis and neuronal death [3]. Despite suffering from HAND, in patients with cART, the classical features of HIV encephalitis and/or brain atrophy often are absent [4]. In fact, the severity of HAND is strongly associated with the loss of synaptic markers in patients on cART [5]. However, the molecular mechanisms underlying HAND-associated synaptic alteration remain largely unclear [6].

One of the major limitations in searching for HAND cures has been the lack of an animal model that recapitulates all of the features of HIV infection in humans [7]. Thus, new animal models to examine how chronicity and aging affect HIV-induced neuropathology are an important current and future need [8]. Previous work has heavily relied on rodent models for the study of HIV pathology [9]. However, results obtained in rodent models are often not easily translated to treatment of humans, given that rodents are not naturally susceptible to HIV infection and do not reflect the in vivo nature of infection [10]. Although nonhuman primates infected with simian immunodeficiency virus (SIV) or genetic chimeras of SIV and HIV have a number of important advantages over small-animal models, they have obvious disadvantages, including considerable genetic variation, that greatly complicate studies using small numbers of animals and high maintenance costs [7]. Moreover, SIV is unable to cause acquired immune deficiency syndrome (AIDS) in its natural host [11,12]. In contrast, feline immunodeficiency virus (FIV) infection in domestic cats represents an animal model of immunodeficiency and shares similarities in pathogenesis with that of HIV in humans [11–13]. Certain strains of FIV can infect the central nervous system (CNS), leading to neurological symptoms similar to those observed in some individuals infected with HIV [13,14]. Importantly, FIV is a naturally occurring virus inducing both AIDS and neurological complications in animal models [15]. Furthermore, the combination of HIV antiretroviral drugs on naturally infected FIV cats in the late phase of the asymptomatic state of the disease significantly reduces viral load, indicating a similar pathogenesis of these viruses [16]. Therefore, FIV infection of cats is an attractive model to study the chronic neuropathogenesis of HAND. Little is known, however, about neuronal mechanisms underpinning overlapping neuropathology between FIV and HIV.

Both HIV and FIV are tropic for lymphocytes and monocytes, utilizing CD4 (HIV) and CD134 (FIV) primary receptors together with the alpha chemokine receptor CXCR4 as a co-receptor to infect cells [17–19]. Even though lentiviral infection in the brain produces cortical and subcortical neuronal loss [20,21], HIV and FIV do not directly infect neurons but instead use a noninfectious interaction between the viral envelope and the neuronal surface [22,23]. Among HIV-induced neuron-damaging products, HIV envelope glycoprotein gp120 is one of the most prominent viral antigens found in the lysates of HIV-infected cells [24]. HIV gp120 indirectly and/or directly interacts with neurons, which enhances excitatory synaptic receptor activity, resulting in synaptic damages, but the mechanisms are not currently understood [25–27]. In neurons, the gp120 interaction with CXCR4 enhances Ca^2+^-regulating systems through NMDA receptors (NMDARs) in the synaptic membrane and inositol trisphosphate receptors (IP3Rs) in the endoplasmic reticulum (ER), resulting in apoptosis [28–34]. In addition, Ca^2+^ fluxes through NMDARs promoting the production of nitric oxide (NO) by neuronal nitric oxide synthase (nNOS), which is tethered by the scaffolding protein postsynaptic density 95 (PSD95) [35–39]. NO subsequently exerts its effects by activating cGMP-dependent protein kinase II (cGKII) through the production of cGMP [40]. Notably, the NMDAR-nNOS-cGK pathway has been implicated in HIV-induced neurotoxicity [41,42]. However, the exact cellular role of the pathway on synaptic dysfunction in HAND has not been determined.

We have shown previously that cGKII can phosphorylate serine 1756 in neuronal IP3Rs and increase ER Ca^2+^ release [43]. cGKII also phosphorylates the AMPA receptor (AMPAR) subunit GluA1, which triggers its synaptic trafficking, a critical step for inducing synaptic plasticity [43,44]. This suggests that cGKII activation is critical for HAND-associated synaptic dysfunction. Here, we demonstrate that FIV envelope glycoprotein, gp95, binds to CXCR4 on the neuronal plasma membrane and utilizes the same pathway as HIV gp120 to significantly increase intracellular Ca^2+^ activity and synaptic activity in neurons. Thus, our results indicate that FIV serves as a model for HAND-associated synaptic hyperexcitation. Most notably, our study reveals the inclusion of cGKII in both FIV gp95 and HIV gp120-induced Ca^2+^ hyperactivity, suggesting that cGKII inhibition may be a novel therapeutic target for HAND.

## Results

### FIV gp95-induced elevation of neuronal Ca^2+^ activity

**Fig 1:**
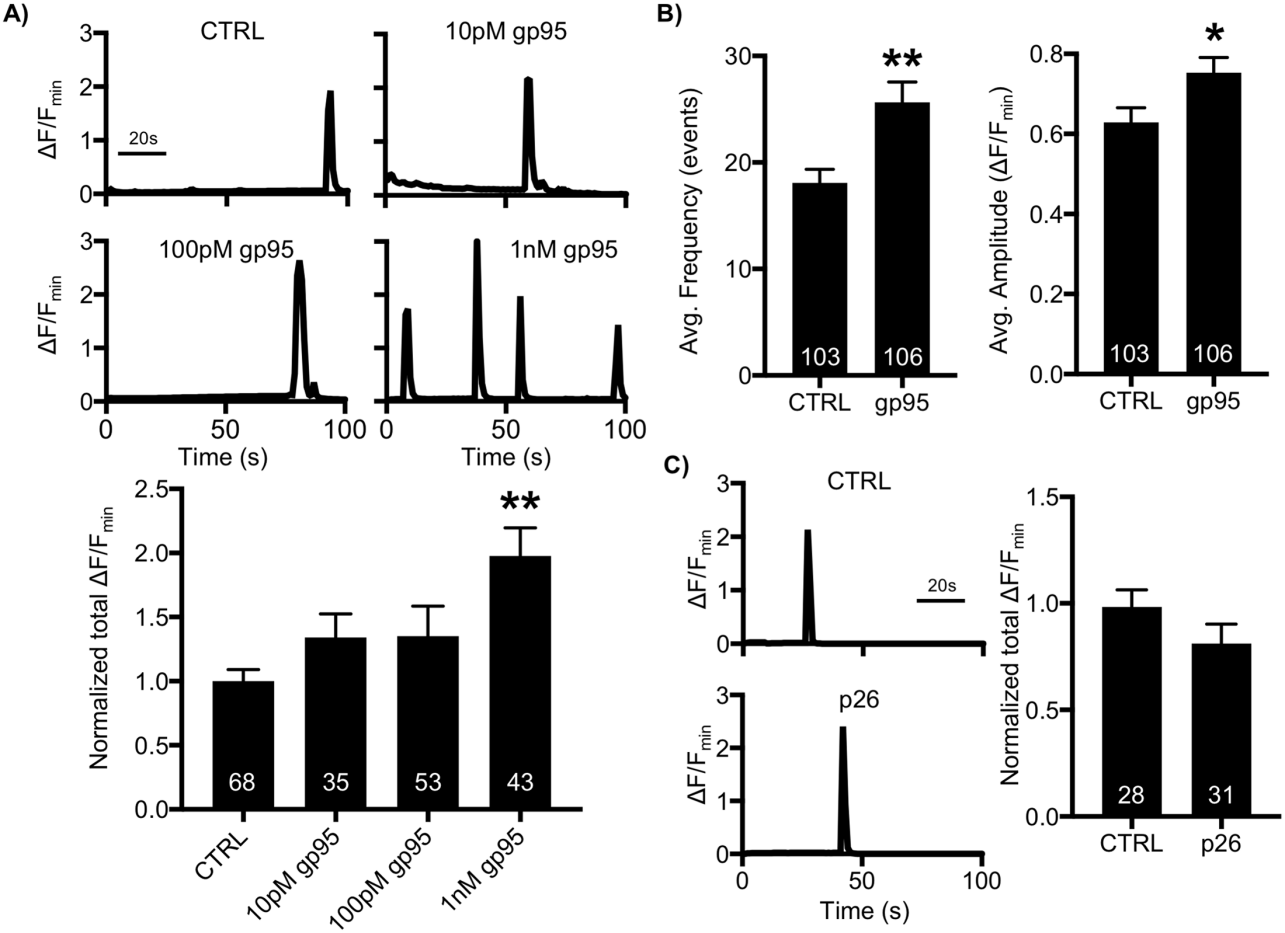
FIV envelope glycoprotein gp95, not capsid protein p26, increases neuronal Ca^2+^ activity. **(A)** Representative traces of GCaMP5 fluorescence intensity and a summary graph of normalized average of total Ca^2+^ activity in control, 10-pM, 100-pM, and 1-nM gp95-treated neurons showing that 1-nM gp95 treatment significantly increases neuronal Ca^2+^ activity (*n* = number of neurons, **p < 0.01, one-way ANOVA, uncorrected Fischer’s LSD, F (3,195) = 5.204, *p* = 0.0018). **(B)** Average frequency and amplitude of Ca^2+^ activity in control and gp95-treated neurons showing that gp95 elevates both frequency and amplitude of Ca^2+^ activity (*n* = number of neurons, *p < 0.05 and **p < 0.01, unpaired two-tailed Student *t* tests). **(C)** FIV capsid protein 700-nM p26 treatment has no effect on neuronal Ca^2+^ activity (*n* = number of cells). A scale bar indicates 20 seconds. FIV, feline immunodeficiency virus; LSD, Least Significant Difference.

Neuronal Ca^2+^ is the second messenger responsible for transmitting depolarization status and synaptic activity [45]. These features make Ca^2+^ regulation a critical process in neurons, and thus altered Ca^2+^ activity in neurons is one of the major contributors to many neurological disorders, including HAND [45]. It has been found that HIV gp120 increases Ca^2+^ dynamics in neurons, contributing to neuronal dysfunction [28–34]. We thus hypothesized that FIV gp95 was able to enhance Ca^2+^ activity in neurons, as seen with HIV gp120. To test this idea, we measured Ca^2+^ activity in cultured mouse hippocampal neurons transfected with GCaMP5, a genetically encoded Ca^2+^ indicator, as described previously (S1 Fig) [43,46,47]. First, we acutely treated 12–14 day in vitro (DIV) neurons with 10-pM, 100-pM, and 1-nM gp95 and determined Ca^2+^ activity immediately after gp95 treatment (Fig 1A). We found active spontaneous Ca^2+^ transients in control cells (CTRL) and neurons treated with gp95 (Fig 1A). However, total Ca^2+^ activity in 1-nM gp95-treated cells was significantly higher than in controls (CTRL, 1 ± 0.09 ΔF/F_min_; gp95, 1.98 ± 0.22 ΔF/F_min_, *p* = 0.0001), confirming that 1-nM gp95 was sufficient to increase neuronal Ca^2+^ activity, while 10-pM (1.34 ± 0.18 ΔF/F_min_) and 100-pM gp95 (1.35 ± 0.23 ΔF/F_min_) slightly elevated Ca^2+^ activity but were not significantly different from control cells (Fig 1A). Importantly, the average frequency (CTRL, 18.05 ± 1.26 events; gp95, 25.65 ± 1.89 events, *p* = 0.001) and amplitude (CTRL, 0.62 ± 0.03 ΔF/F_min_; gp95, 0.73 ± 0.03 ΔF/F_min_, *p* = 0.018) were significantly elevated in gp95-treated neurons (Fig 1B). Next, we treated neurons with 700-nM FIV p26 capsid protein and found that p26 had no effect on neuronal Ca^2+^ activity (Fig 1C), suggesting that Ca^2+^ hyperactivity is caused selectively by FIV gp95.

### Cellular pathway of gp95-induced Ca^2+^ overactivation

**Fig 2:**
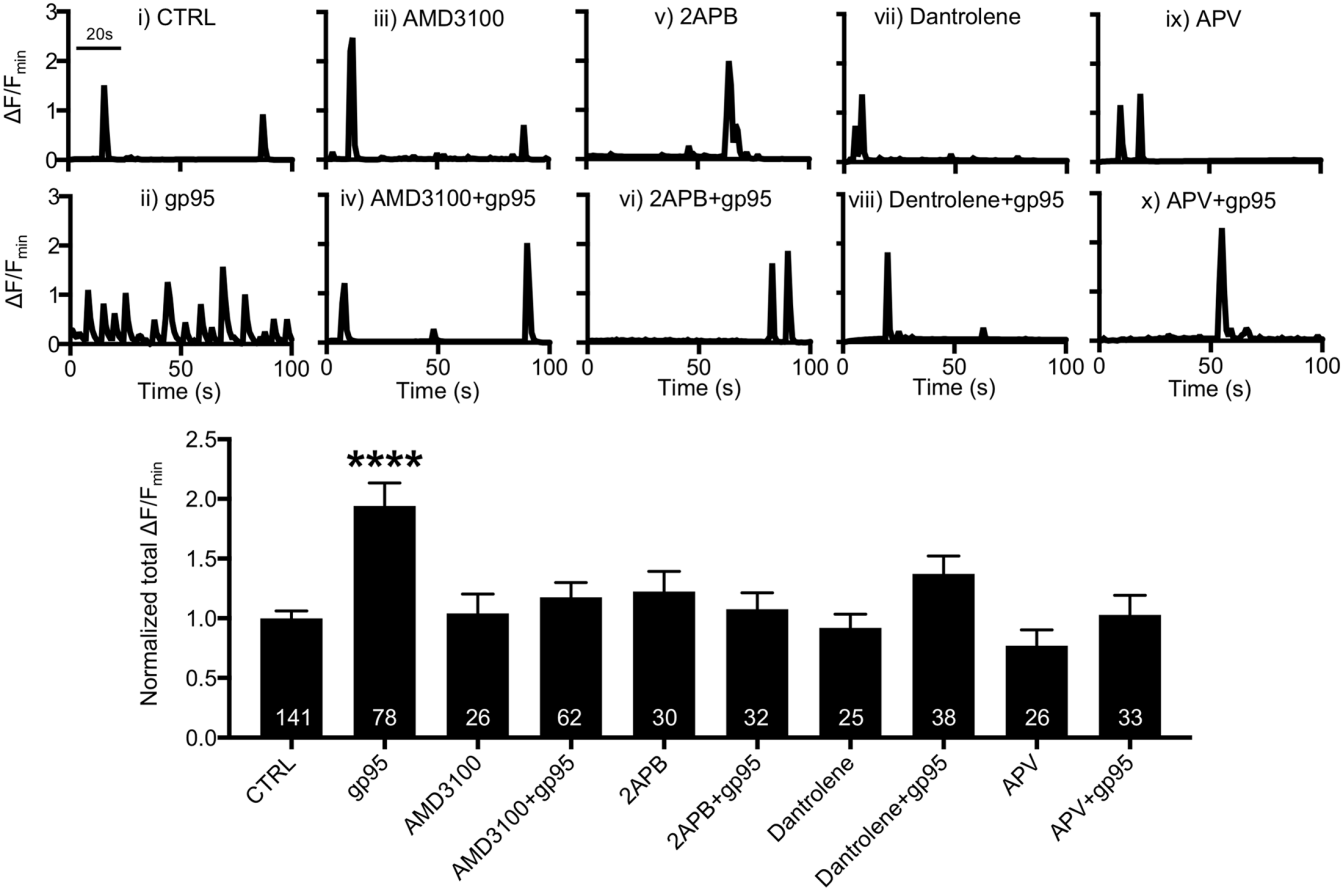
Cellular pathway of gp95-induced Ca^2+^ hyperactivity. Representative traces of GCaMP5 fluorescence intensity and a summary graph of normalized average of total Ca^2+^ activity in **(i)** control neurons and neurons treated with **(ii)** 1-nM gp95, **(iii)** 200-nM AMD3100, **(iv)** 200-nM AMD3100 and 1-nM gp95, **(v)** 25-μM 2APB, **(vi)** 25-μM 2APB and 1-nM gp95, **(vii)** 10-μM Dantrolene, **(viii)** 10-μM Dantrolene and 1-nM gp95, **(ix)** 25-μM DL-APV, and **(x)** 25-μM DL-APV and 1-nM gp95, showing that the gp95-induced elevation of neuronal Ca^2+^ activity is dependent on CXCR4, IP3Rs, RyRs, and NMDARs (*n* = number of neurons, ****p < 0.0001, one-way ANOVA, uncorrected Fischer’s LSD, F (9,481) = 6.289). A scale bar indicates 20 seconds. AMD3100, bicyclam derivative plerixafor hydrochloride; IP3R, inositol triphosphate receptor; LSD, Least Significant Difference; NMDAR, NMDA receptor; RyR, Ryanodine receptor.

HIV gp120 interacts with CXCR4 on neurons, subsequently elevating intracellular Ca^2+^ through mobilizing ER Ca^2+^ [48], as well as by NMDARs [32]. Similarly, FIV gp95 also interacts with CXCR4 [18,49,50]. We thus hypothesized that HIV gp120 and FIV gp95 shared a core Ca^2+^ hyperexcitation pathway in neurons. Using GCaMP5, we confirmed that 1-nM gp95 was sufficient to increase Ca^2+^ activity compared with control neurons (CTRL, 1 ± 0.06 ΔF/F_min_; gp95, 1.94 ± 0.19; ΔF/F_min_ *p* < 0.0001) (Fig 2i and 2ii). We used 200-nM bicyclam derivative plerixafor hydrochloride (AMD3100) to block the interaction between gp95 and CXCR4 and identified that AMD3100 treatment was sufficient to inhibit gp95-induced Ca^2+^ hyperactivity (1.18 ± 0.12 ΔF/F_min_, *p* < 0.0001) (Fig 2iv), while 200-nM AMD3100 alone had no effect on Ca^2+^ activity (1.04 ± 0.16 ΔF/F_min_) (Fig 2iii). This suggests that CXCR4 is required for the gp95 effects on Ca^2+^ activity. HIV gp120 binds to CXCR4, promoting ER Ca^2+^ release through a rapid hydrolysis of phospholipase C to generate IP3, which then activates ER Ca^2+^ channels, IP3Rs [51–53]. To confirm whether gp95 increased Ca^2+^ activity via IP3Rs, we treated neurons with 25-μM 2APB, an IP3R blocker and found that 2APB blocked the gp95-induced elevation of Ca^2+^ activity (1.08 ± 0.13 ΔF/F_min_, *p* < 0.0001) (Fig 2vi). However, 25-μM 2APB alone was unable to alter GCaMP5 activity (1.22 ± 0.17 ΔF/F_min_) (Fig 2v). Ryanodine receptors (RyRs) are another ER-associated Ca^2+^ channel [54]. To test whether RyRs were involved in gp95-induced Ca^2+^ hyperactivity, we treated neurons with 10-μM dantrolene, a RyR blocker, and found that inhibition of RyRs also abolished gp95 effects (1.37 ± 0.15 ΔF/F_min_, *p* = 0.0047) (Fig 2viii). Treatment with 10-μM dantrolene alone had no effect on Ca^2+^ activity (0.92 ± 0.11 ΔF/F_min_) (Fig 2vii). This suggests that the gp95-induced elevation of Ca^2+^ activity is dependent on ER Ca^2+^ release. Finally, we treated neurons with 1-nM gp95 and 50-μM DL-APV, a NMDAR antagonist, and found that 50-μM DL-APV completely inhibited GCaMP5 activity in both control and gp95-treated cells (DL-APV, 0.13 ± 0.04 ΔF/F_min_, p<0.0001; DL-APV+gp95, 0.1 ± 0.06 ΔF/F_min_, *p* < 0.0001) (S2 Fig). However, a lower dose of DL-APV (25 μM) was unable to affect basal Ca^2+^ activity (0.77 ± 0.13 ΔF/F_min_) (Fig 2ix). We thus used 25-μM DL-APV to avoid the inhibition of basal Ca^2+^ activity and directly assay the gp95 effects. Importantly, 25-μM DL-APV was sufficient to block gp95 effects (1.03 ± 0.16 ΔF/F_min_, *p* < 0.0001) (Fig 2x). This suggests that synaptic NMDAR activity is critical for gp95-induced Ca^2+^ overexcitation. Taking these data together, we confirm that gp95 interacts with CXCR4 and activates ER Ca^2+^ channels and synaptic NMDARs, enhancing neuronal Ca^2+^ activity, similar to HIV gp120.

### Gp95-induced Ca^2+^ hyperexcitation is dependent on cGKII-mediated IP3R activation

**Fig 3:**
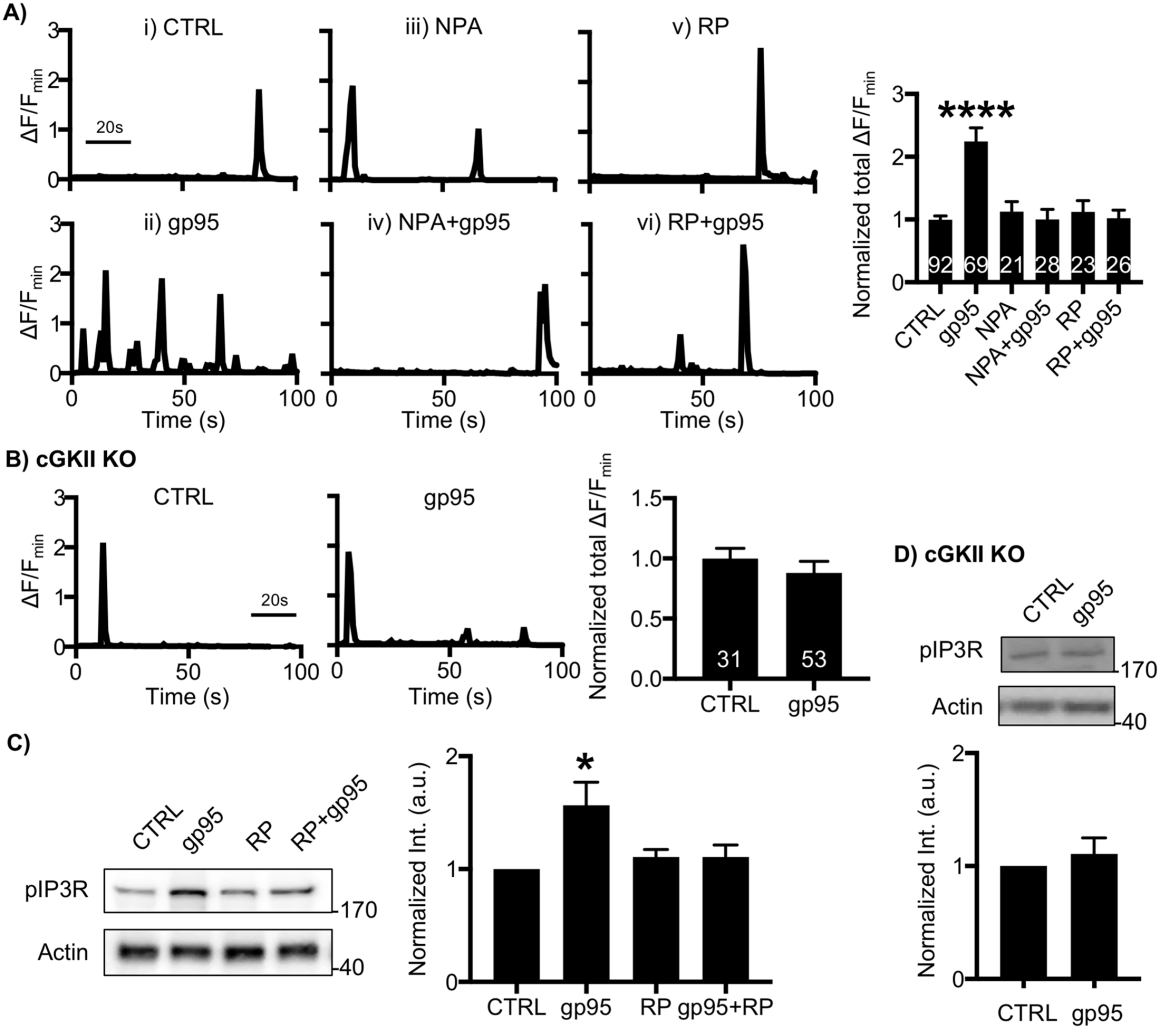
Gp95-induced Ca^2+^ hyperactivity is mediated by nNOS-cGKII activation. **(A)** Representative traces of GCaMP5 fluorescence intensity and a summary graph of normalized average of total Ca^2+^ activity in **(i)** control neurons and neurons treated with **(ii)** 1-nM gp95, **(iii)** 2-μM NPA, **(iv)** 2-μM NPA and 1-nM gp95, **(v)** 1-μM RP, and **(vi)** 1-μM RP and 1-nM gp95 showing that nNOS activity and cGKII activity are required for gp95-induced Ca^2+^ hyperactivity (*n* = number of neurons, ****p < 0.0001, one-way ANOVA, uncorrected Fischer’s LSD, F (5,253) = 12.38). A scale bar indicates 20 seconds. **(B)** gp95 is unable to induce neurotoxic effects when the *cGKII* gene is deleted (*n* = number of neurons). A scale bar indicates 20 seconds. **(C)** Representative immunoblots and quantitative analysis of pIP3R(S1756) levels in each condition showing that gp95 treatment is able to increase pIP3Rs, which are dependent on cGKII activity (*n* = 4 experiments, *p < 0.05, one-way ANOVA, uncorrected Fischer’s LSD, F (3,20) = 3.795, *p* = 0.0264). **(D)** Representative immunoblots and quantitative analysis of pIP3R(S1756) levels in WT and cGKII KO neurons showing that gp95 treatment has no effect on IP3R phosphorylation (*n* = 4 experiments). cGKII, cGMP-dependent protein kinase II; IP3R, inositol triphosphate receptor; KO, knockout; LSD, Least Significant Difference; nNOS, neuronal nitric oxide synthase; NPA, *N*^ω^-Propyl-L-arginine hydrochloride.

Ca^2+^ flux through the NMDAR-nNOS pathway activates cGKII by the production of cGMP [40]. We thus hypothesized that gp95 stimulated the NMDAR-nNOS pathway, leading to the activation of cGKII to phosphorylate IP3Rs, resulting in enhanced neuronal Ca^2+^ dynamics. Indeed, we found that inhibition of nNOS blocked gp95 effects on Ca^2+^ activity by treating neurons with 2-μM *N*^ω^-Propyl-L-arginine hydrochloride (NPA), a nNOS inhibitor (CTRL, 1 ± 0.06 ΔF/F_min_; gp95, 2.24 ± 0.21 ΔF/F_min_, *p* < 0.0001; NPA+gp95, 0.99 ± -0.16 ΔF/F_min_, p<0.0001) (Fig 3Ai, 3Aii and 3Aiv). Next, we tested the possibility that cGKII was a downstream effector of gp95. We found that gp95 was unable to increase Ca^2+^ activity when cGKII activity was blocked by treating neurons with 1-μM Rp8-Br-PET-cGMPS (RP), a cGKII inhibitor (1.02 ± 0.13 ΔF/F_min_, *p* < 0.0001) (Fig 3Avi). Notably, NPA or RP itself was unable to affect GCaMP activity (NPA, 1.12 ± 0.16 ΔF/F_min_; RP, 1.12 ± 0.16 ΔF/F_min_) (Fig 3Aiii and 3Av). We additionally cultured neurons from cGKII knockout (KO) animals as described previously [43] and confirmed that 1-nM gp95 had no effect on Ca^2+^ dynamics in KO neurons (CTRL, 1 ± 0.08 ΔF/F_min_; gp95, 0.88 ± 0.09 ΔF/F_min_, *p* = 0.39) (Fig 3B). This suggests that cGKII activation is required for gp95-induced Ca^2+^ hyperactivity. Significantly, cGKII can phosphorylate serine 1756 in neuronal IP3Rs (pIP3Rs), increasing Ca^2+^ currents in neuronal tissues [43,55]. We thus tested whether neuronal IP3R phosphorylation was altered by gp95 treatment. As expected, 1-nM gp95 treatment for 1 hour was sufficient to increase pIP3Rs, while RP by itself had no effect on IP3R phosphorylation (Fig 3C), consistent with elevated Ca^2+^ activity (Fig 3A). However, pharmacological inhibition of cGKII activity or genetic deletion of the *cGKII* gene abolished the gp95-mediated elevation of pIP3Rs (Fig 3C and 3D). In summary, gp95 interacts with CXCR4, promoting IP3 production and NMDAR-nNOS-cGKII-mediated IP3R phosphorylation, resulting in ER Ca^2+^ release.

### Gp95 increases surface expression of the AMPAR GluA1 subunit via cGKII activation

**Fig 4:**
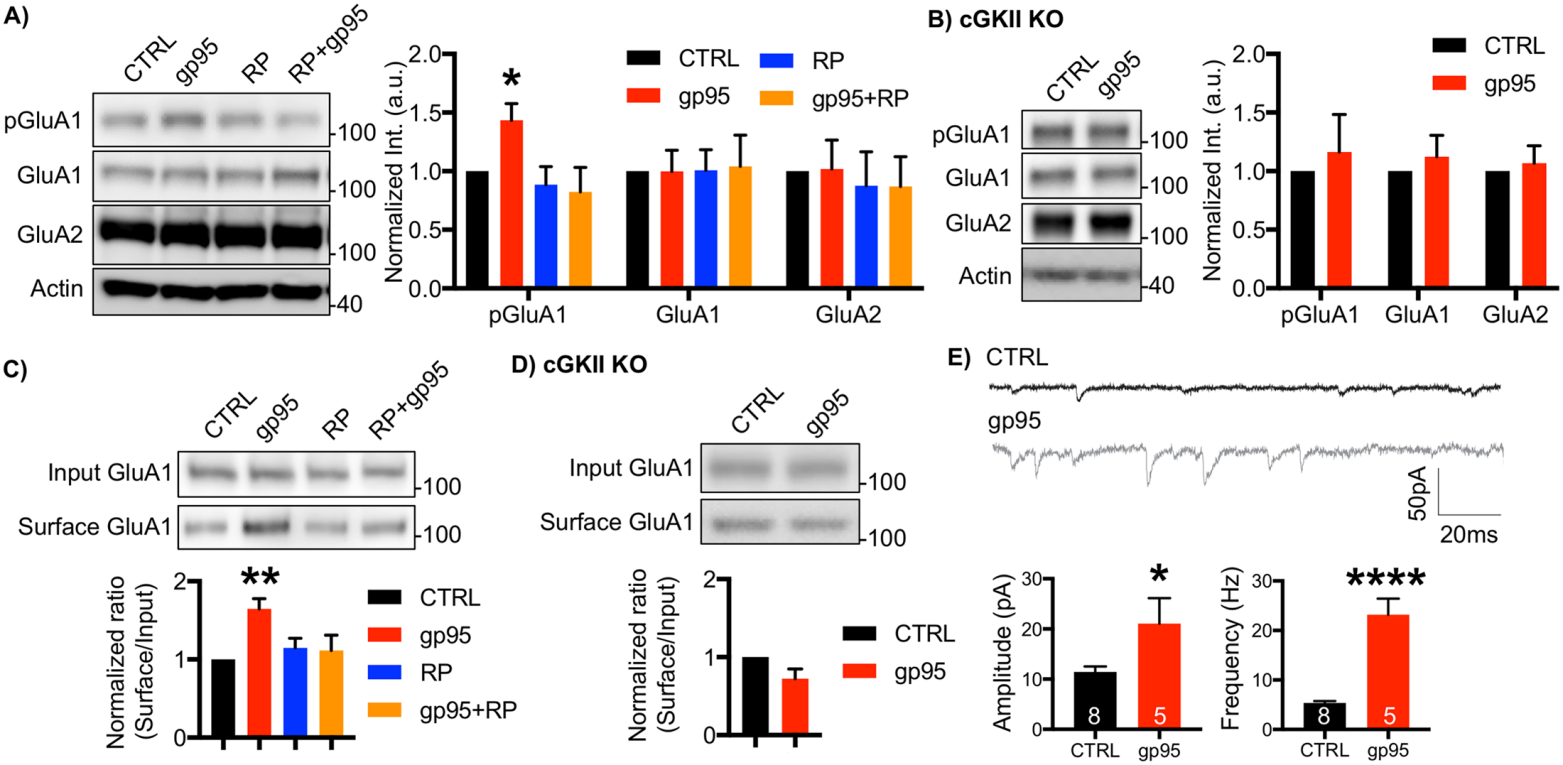
Gp95 increases surface expression of the AMPAR GluA1 subunit via cGKII activation. **(A)** Representative immunoblots and quantitative analysis of the synaptosome fraction from cultured cortical neurons in each condition showing that gp95 is capable of elevating GluA1 S845 phosphorylation (pGluA1), which is mediated by cGKII (*n* = 3 experiments, *p < 0.05, one-way ANOVA, uncorrected Fischer’s LSD, F (3,22) = 3.884, *p* = 0.0228). **(B)** Representative immunoblots and quantitative analysis of the synaptosome fraction from cultured WT and cGKII KO cortical neurons showing that gp95 is unable to increase pGluA1 in cGKII KO neurons (*n* = 5 experiments). **(C)** Representative immunoblots and quantitative analysis of surface biotinylation in each condition showing that gp95 is able to increase surface GluA1 via cGKII activation (*n* = 6 experiments, *p < 0.05, one-way ANOVA, uncorrected Fischer’s LSD, F (3,20) = 3.839, *p* = 0.0254). **(D)** Representative immunoblots and quantitative analysis of surface biotinylation in WT and KO hippocampal neurons showing that cGKII is required for gp95-induced GluA1 surface trafficking (*n* = 5 experiments). **(E)** Representative traces of mEPSC recordings in control and gp95-treated neurons showing average mEPSC amplitude and frequency are significantly increased by gp95 treatment (*n* = number of neurons, *p < 0.05 and ****p < 0.0001, unpaired two-tailed Student *t* tests). AMPAR, AMPA receptor; cGKII, cGMP-dependent protein kinase II; KO, knockout; LSD, Least Significant Difference; mEPSC, miniature excitatory postsynaptic current.

cGKII mediates phosphorylation of serine 845 of GluA1 (pGluA1), important for activity-dependent trafficking of GluA1-containing AMPARs, and increases the level of extrasynaptic receptors [43,44]. Moreover, cGKII-mediated GluA1 phosphorylation is critical for hippocampal long-term potentiation (LTP) and learning and memory [43,44]. As gp95 was sufficient to induce cGKII activation (Fig 3), we hypothesized that gp95-induced cGKII activation increased GluA1 phosphorylation, which led to enhanced AMPAR-mediated synaptic activity. To test this idea, we first biochemically measured GluA1 phosphorylation levels when gp95 was applied. Mouse cultured cortical neurons were treated with 1-nM gp95 for 1 hour, and synaptosomes were isolated as described previously [47] (S3 Fig). We found that gp95 treatment was sufficient to increase GluA1 phosphorylation, while total GluA1 and GluA2 levels were not affected (Fig 4A). To confirm whether such an increase was dependent on cGKII, we treated neurons with gp95 and 1-μM RP for 1 hour and measured pGluA1 levels. We revealed that inhibition of cGKII activity abolished the gp95 effects, while RP by itself was unable to affect AMPAR synaptic expression (Fig 4A). To further confirm the role of cGKII in the elevation of GluA1 phosphorylation, we used cGKII KO neurons and found that gp95 treatment had no effect on GluA1 phosphorylation in KO neurons (Fig 4B). Given that GluA1 phosphorylation promotes AMPAR surface expression, we next measured surface GluA1 levels by biotinylation after 1-nM gp95 was applied for 1 hour. We found that gp95 treatment increased surface GluA1 levels, which was blocked by pharmacological and genetic inhibition of cGKII (Fig 4C and 4D). Notably, cGKII inhibition by itself had no effect on GluA1 surface expression (Fig 4C). Furthermore, gp95 treatment was unable to alter GluA2 and NMDAR subunit NR1 surface expression (S4 Fig). This suggests that cGKII is required for gp95-induced GluA1 up-regulation. To further confirm whether such an increase in surface expression of AMPARs elevates AMPAR-mediated synaptic transmission, we measured miniature excitatory postsynaptic currents (mEPSCs) in DIV14-17 cultured mouse hippocampal neurons (Fig 4E). We found that acute treatment of 1-nM gp95 significantly increased both average mEPSC amplitude (CTRL, 11.45 ± 1.07 pA; gp95, 21.07 ± 5.06, *p* = 0.04) and frequency (CTRL, 5.35 ± 0.37 Hz; gp95, 23.17 ± 3.29, *p* < 0.0001) (Fig 4E). This suggests that gp95-induced activation of cGKII increases surface expression of AMPARs, contributing to enhanced synaptic transmission.

### Activity-dependent gp95 effects on AMPAR-mediated Ca^2+^ hyperactivity

**Fig 5:**
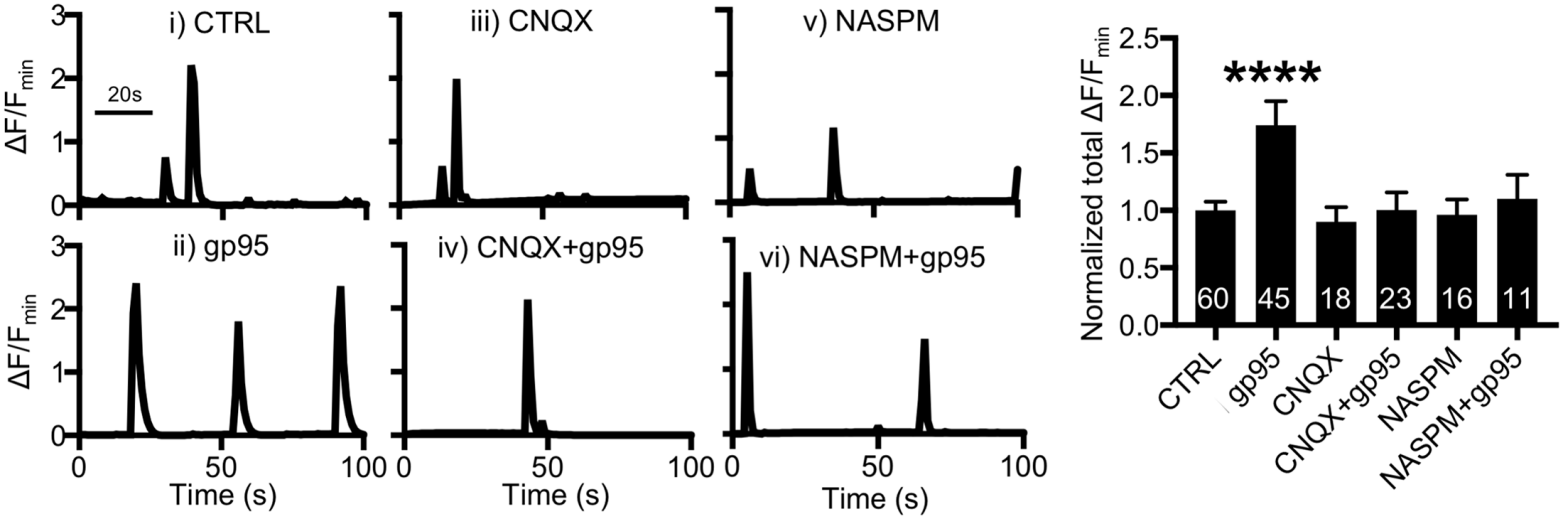
Activity-dependent gp95 effects on AMPAR-mediated Ca^2+^ hyperactivity. Representative traces of GCaMP5 fluorescence intensity and a summary graph of normalized average of total Ca^2+^ activity in **(i)** control neurons and neurons treated with **(ii)** 1-nM gp95, **(iii)** 10-μM CNQX, **(iv)** 10-μM CNQX and 1-nM gp95, **(v)** 20-μM NASPM, and **(vi)** 20-μM NASPM and 1-nM gp95, showing that CP-AMPARs are required for gp95-induced Ca^2+^ hyperactivity (*n* = number of neurons, ****p < 0.0001, one-way ANOVA, uncorrected Fischer’s LSD, F (7,179) = 12.1). A scale bar indicates 20 seconds. AMPAR, AMPA receptor; CNQX, 6-Cyano-7-nitroquinoxaline-2,3-dione; CP-AMPAR, Ca^2+^-permeable AMPAR; LSD, Least Significant Difference; NASPM, 1-naphthyl acetyl spermine.

We next examined whether an elevation of AMPAR function was responsible for gp95-induced Ca^2+^ hyperactivity. We carried out Ca^2+^ imaging in the presence of an AMPAR inhibitor, 6-Cyano-7-nitroquinoxaline-2,3-dione (CNQX) (Fig 5). Treatment with 30-μM CNQX significantly blocked GCaMP5 activity in both control and gp95-treated neurons (S5 Fig). However, 10-μM CNQX alone had no effect on Ca^2+^ activity (0.9 ± 0.12 ΔF/F_min_) (Fig 5iii); thus, we used 10-μM CNQX to avoid the inhibition of basal Ca^2+^ activity and directly assay the gp95 effects. Notably, the gp95-induced increase in Ca^2+^ activity (CTRL, 1 ± 0.07 ΔF/F_min_; gp95, 1.74 ± 0.21 ΔF/F_min_, *p* < 0.0001) (Fig 5i and 5ii) was completely inhibited by treating neurons with a lower dose of CNQX (1.00 ± 0.15 ΔF/F_min_, *p* = 0.009) (Fig 5iv), suggesting that gp95-induced elevated AMPAR activity is required for Ca^2+^ hyperactivity. Notably, there are two general types of AMPARs formed through combination of the subunits, Ca^2+^-impermeable GluA2-containing and Ca^2+^-permeable, GluA2-lacking/GluA1-containing receptors [56]. Ca^2+^-permeable AMPARs (CP-AMPARs) are generally sensitive to polyamine block [57]. As we found that gp95 selectively elevated GluA1 surface expression (Fig 4 and S4 Fig), these AMPARs could be CP-AMPARs. We thus used 20-μM 1-naphthyl acetyl spermine (NASPM), an antagonist of CP-AMPARs, to determine if CP-AMPARs were responsible for the gp95-mediated Ca^2+^ hyperactivity. We found that 20-μM NASPM treatment was sufficient to block the gp95 effects on Ca^2+^ activity (1.10 ± 0.20 ΔF/F_min_, *p* = 0.02) (Fig 5vi), while NASPM alone was able to alter Ca^2+^ dynamics (0.96 ± 0.13 ΔF/F_min_) (Fig 5v). To further test whether the gp95 effects were dependent on neuronal activity, we treated neurons with 1-μM tetrodotoxin (TTX) and found that TTX treatment completely blocked GCaMP5 activity in both control and gp95-treated neurons (S6A Fig). Taking these data together, FIV gp95-induced Ca^2+^ hyperexcitation is dependent on AMPAR- and NMDAR-mediated neuronal activity.

### FIV gp95-induced Ca^2+^ hyperactivity in cultured feline hippocampal neurons

**Fig 6:**
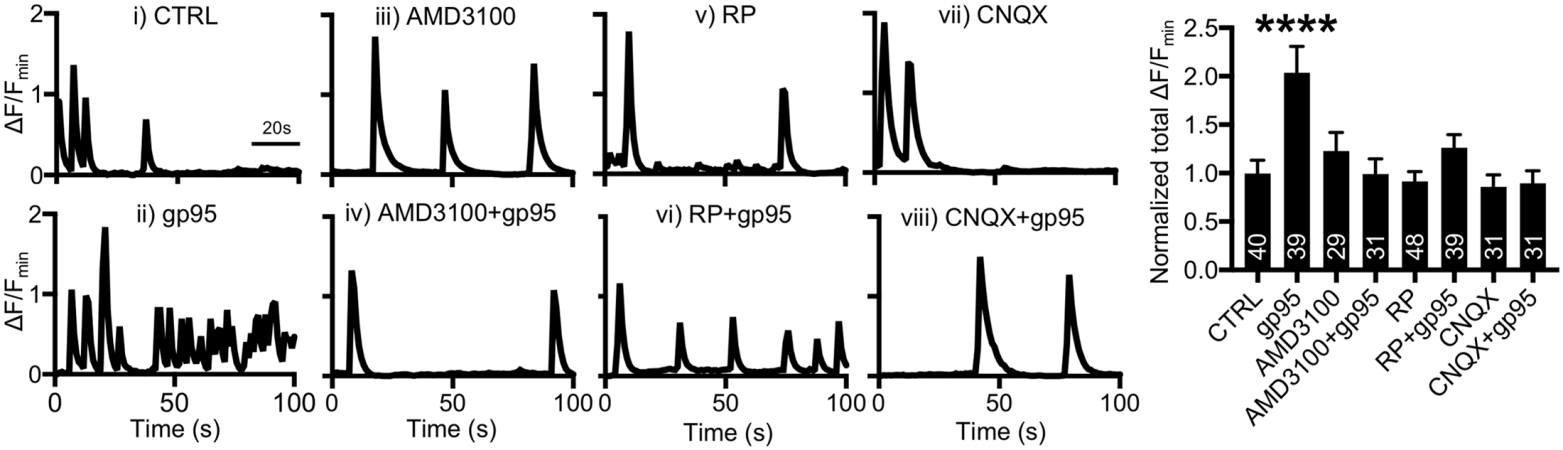
Gp95-induced Ca^2+^ hyperactivity in feline cultured hippocampal neurons. Representative traces of GCaMP5 fluorescence intensity and a summary graph of normalized average of total Ca^2+^ activity in **(i)** control neurons and neurons treated with **(ii)** 1-nM gp95, **(iii)** 200-nM AMD3100, **(iv)** 200-nM AMD3100 and 1-nM gp95, **(v)** 10-μM RP, **(vi)** 10-μM RP and 1-nM gp95, **(vii)** 10-μM CNQX, and **(viii)** 10-μM CNQX and 1-nM gp95, showing that the gp95-induced Ca^2+^ hyperactivity in cat hippocampal neurons is dependent on CXCR4, cGKII, and AMPARs (*n* = number of neurons, ****p < 0.0001, one-way ANOVA, uncorrected Fischer’s LSD, F (7,260) = 5.296). A scale bar indicates 20 seconds. AMD3100, bicyclam derivative plerixafor hydrochloride; AMPAR, AMPA receptor; cGKII, cGMP-dependent protein kinase II; CNQX, 6-Cyano-7-nitroquinoxaline-2,3-dione; LSD, Least Significant Difference.

We next examined gp95-induced Ca^2+^ overactivation in cultured feline neurons. We first treated 14–16 DIV cultured feline hippocampal neurons with 1-nM gp95 and determined Ca^2+^ activity. Consistent with the findings in cultured mouse neurons (Fig 1A), total Ca^2+^ activity in 1-nM gp95-treated feline cells was significantly higher than in controls (CTRL, 1 ± 0.13 ΔF/F_min_; gp95, 2.03 ± 0.27 ΔF/F_min_, *p* < 0.0001) (Fig 6i and 6ii), indicating that 1-nM gp95 was also capable of increasing neuronal Ca^2+^ activity in feline neurons. To determine whether CXCR4 was involved in the gp95 effect on cat neurons, we treated neurons with 200-nM AMD3100, which was sufficient to inhibit gp95-induced Ca^2+^ hyperactivity (0.99 ± 0.15 ΔF/F_min_, *p* < 0.0001) (Fig 6iv). We also treated neurons with 10-μM RP and revealed that inhibition of cGKII was sufficient to block gp95 effects (1.26 ± 0.12 ΔF/F_min_, *p* < 0.0001) (Fig 6vi). This confirms that cGKII activation is also required for gp95-induced Ca^2+^ hyperactivity in feline neurons. We finally inhibited AMPAR function by treating neurons with 10-μM CNQX and revealed that gp95 was unable to elicit Ca^2+^ overactivation when AMPARs were inhibited (0.89 ± 0.13 ΔF/F_min_, *p* < 0.0001) (Fig 6viii). Notably, inhibitors by themselves had no effect on Ca^2+^ activity in feline neurons as well (AMD3100, 1.22 ± 0.19 ΔF/F_min_; RP, 0.91 ± 0.1 ΔF/F_min_; CNQX, 0.85 ± 0.12 ΔF/F_min_) (Fig 6iii, 6v and 6vii). This suggests that FIV gp95 is also sufficient to induce activity-dependent Ca^2+^ hyperexcitation in cultured feline neurons.

### cGKII activation is required for HIV gp120 and SDF-1-induced Ca^2+^ hyperactivity

**Fig 7:**
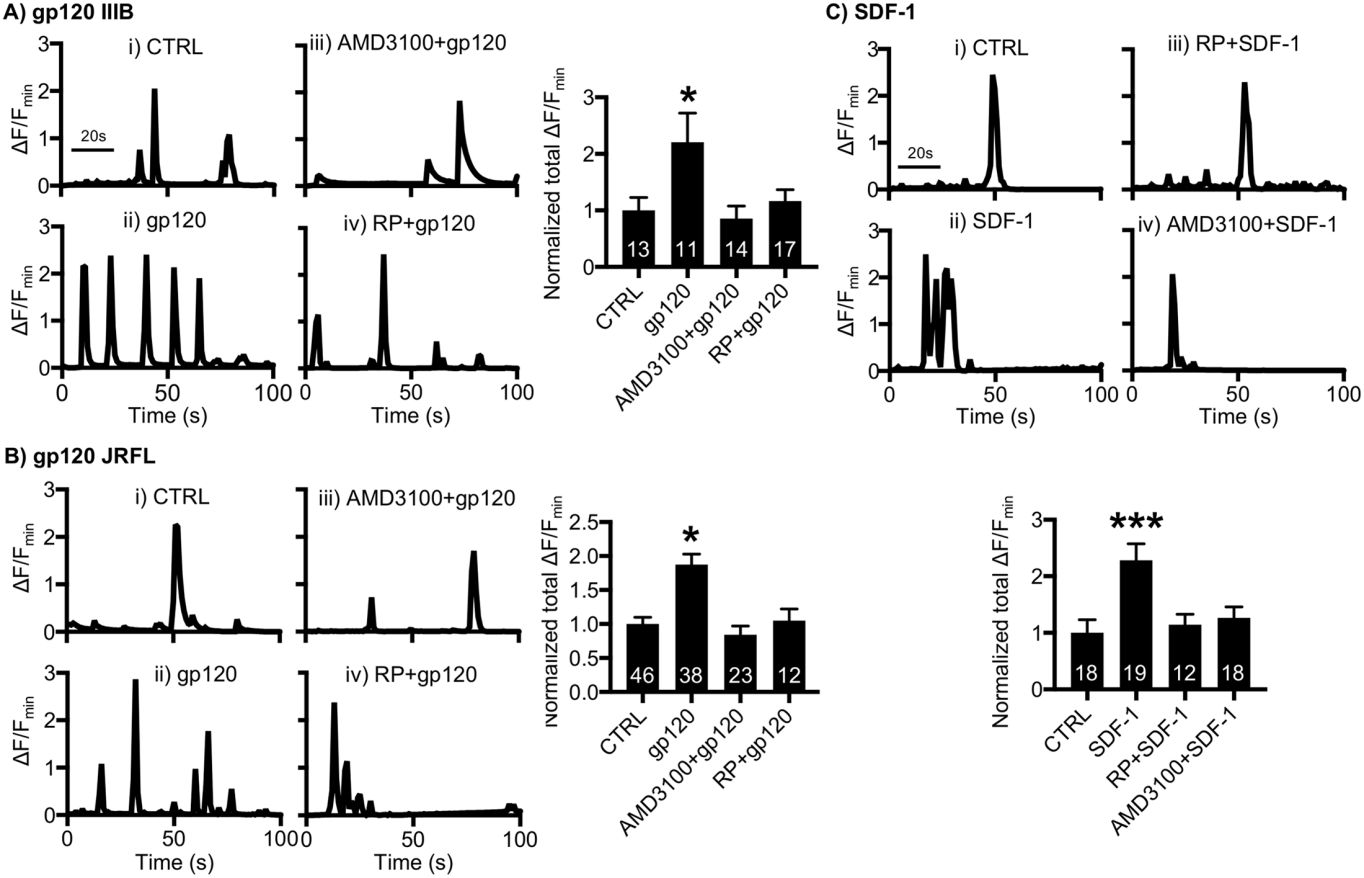
cGKII activation is required for HIV gp120 and SDF-1-induced Ca^2+^ hyperactivity. **(A)** Representative traces of GCaMP5 fluorescence intensity and a summary graph of normalized average of total Ca^2+^ activity in **(i)** control neurons and neurons treated with **(ii)** 1-nM gp120 (IIIB), **(iii)** 200-nM AMD3100 and 1-nM gp120 (IIIB), and **(iv)** 1-μM RP and 1-nM gp120 (IIIB), showing that an increase in Ca^2+^ activity by 1-nM CXCR4-tropic gp120 (IIIB) treatment is dependent on CXCR4 and cGKII (*n* = number of neurons, *p < 0.05, one-way ANOVA, uncorrected Fischer’s LSD, F (3,51) = 3.936, *p* = 0.0133). A scale bar indicates 20 seconds. **(B)** Representative traces of GCaMP5 fluorescence intensity and a summary graph of normalized average of total Ca^2+^ activity in **(i)** control neurons and neurons treated with **(ii)** 1-nM gp120 (JRFL), **(iii)** 200-nM AMD3100 and 1-nM gp120 (JRFL), and **(iv)** 1-μM RP and 1-nM gp120 (JRFL), showing that an increase in Ca^2+^ activity by 1-nM CCR5-tropic gp120 (JRFL) treatment is dependent on CXCR4 and cGKII (*n* = number of neurons, *p < 0.05, one-way ANOVA, uncorrected Fischer’s LSD, F (3,115) = 3.903, *p* = 0.0107). A scale bar indicates 20 seconds. **(C)** Representative traces of GCaMP5 fluorescence intensity and a summary graph of normalized average of total Ca^2+^ activity in **(i)** control neurons and neurons treated with **(ii)** 20-nM SDF-1, **(iii)** 1-μM RP and 20-nM SDF-1, and **(iv)** 200-nM AMD3100 and 20-nM SDF-1, showing that 20-nM SDF-1 induces cGKII- and CXCR4-dependent Ca^2+^ overactivation (*n* = number of neurons, ***p < 0.001, one-way ANOVA, uncorrected Fischer’s LSD, F (3,63) = 6.234, *p* = 0.0009). A scale bar indicates 20 seconds. AMD3100, bicyclam derivative plerixafor hydrochloride; cGKII, cGMP-dependent protein kinase II; LSD, Least Significant Difference; SDF-1, stromal cell-derived factor-1.

We finally investigated whether cGKII activation was required for HIV gp120-induced Ca^2+^ hyperactivity. We treated 12–14 DIV cultured mouse hippocampal neurons with 1-nM CXCR4-tropic gp120 (IIIB) and determined Ca^2+^ activity (Fig 7A). As expected, total Ca^2+^ activity in gp120-treated cells was significantly higher than in controls (CTRL, 1 ± 0.22 ΔF/F_min_; gp120 (IIIB), 2.20 ± 0.51 ΔF/F_min_, *p* = 0.0075) (Fig 7Ai and 7Aii), consistent with previous findings [27, 58]. Furthermore, 200-nM AMD3100 treatment was sufficient to inhibit gp120-induced Ca^2+^ hyperactivity (0.85 ± 0.22 ΔF/F_min_, *p* = 0.0025) (Fig 7Aiii), confirming that CXCR4 is required for gp120-induced Ca^2+^ overexcitation. Importantly, neurons treated with 1-μM RP and gp120 together exhibited no increased Ca^2+^ activity (1.16 ± 0.20 ΔF/F_min_, *p* = 0.0141) (Fig 7Aiv), suggesting that cGKII activity is critical for CXCR4-tropic gp120 (IIIB)-induced aberrant Ca^2+^ activation. Unlike T-cell tropic viruses that use CXCR4, macrophage-tropic HIV uses a chemokine receptor CCR5 as a co-receptor [59]. We next treated neurons with 1-nM CCR5-tropic gp120 (JRFL) and found that gp120 (JRFL) was also able to induce Ca^2+^ overactivation (CTRL, 1 ± 0.09 ΔF/F_min_; gp120 (JRFL), 1.87 ± 0.15 ΔF/F_min_, *p* < 0.0001) (Fig 7Bi and 7Bii), which was abolished by RP treatment (1.05 ± 0.16 ΔF/F_min_, *p* = 0.0014) (Fig 7Biv). This suggests that cGKII activation is required for CXCR4 and CCR5-tropic gp120-indcued Ca^2+^ hyperactivity. We further examined whether CXCR4 inhibition affected the CCR5-tropic gp120 (JRFL) effects on Ca^2+^ activity by treating neurons with 1-nM gp120 (JRFL) and 200-nM AMD3100 together. We found that AMD3100 treatment was also able to block gp120 (JRFL)-induced Ca^2+^ hyperexcitation (0.84 ± 0.13 ΔF/F_min_, *p* < 0.0001) (Fig 7Biii). This suggests that there is cross talk between the two chemokine receptors. Additionally, the natural agonist of CXCR4, stromal cell-derived factor-1 (SDF-1), is by itself neurotoxic [60,61]. We thus examined whether SDF-1 was sufficient to induce cGKII-dependent Ca^2+^ overactivation. Notably, 20-nM SDF-1 treatment was able to increase neuronal Ca^2+^ activity (CTRL, 1 ± 0.23 ΔF/F_min_; SDF-1, 2.28 ± 0.29 ΔF/F_min_, *p* = 0.0002) (Fig 7Ci and 7Cii), which was abolished by RP or AMD3100 treatment (RP, 1.14 ± 0.18 ΔF/F_min_, *p* = 0.0029; AMD3100, 1.26 ± 0.19 ΔF/F_min_, *p* = 0.0028) (Fig 7Ciii and 7Civ), confirming that CXCR4 stimulation is sufficient to induce cGKII-dependent Ca^2+^ overactivation. In summary, we confirm that both FIV gp95 and HIV gp120 interact with CXCR4 and subsequently promote cGKII-mediated Ca^2+^ hyperexcitation.

## Discussion

**Fig 8:**
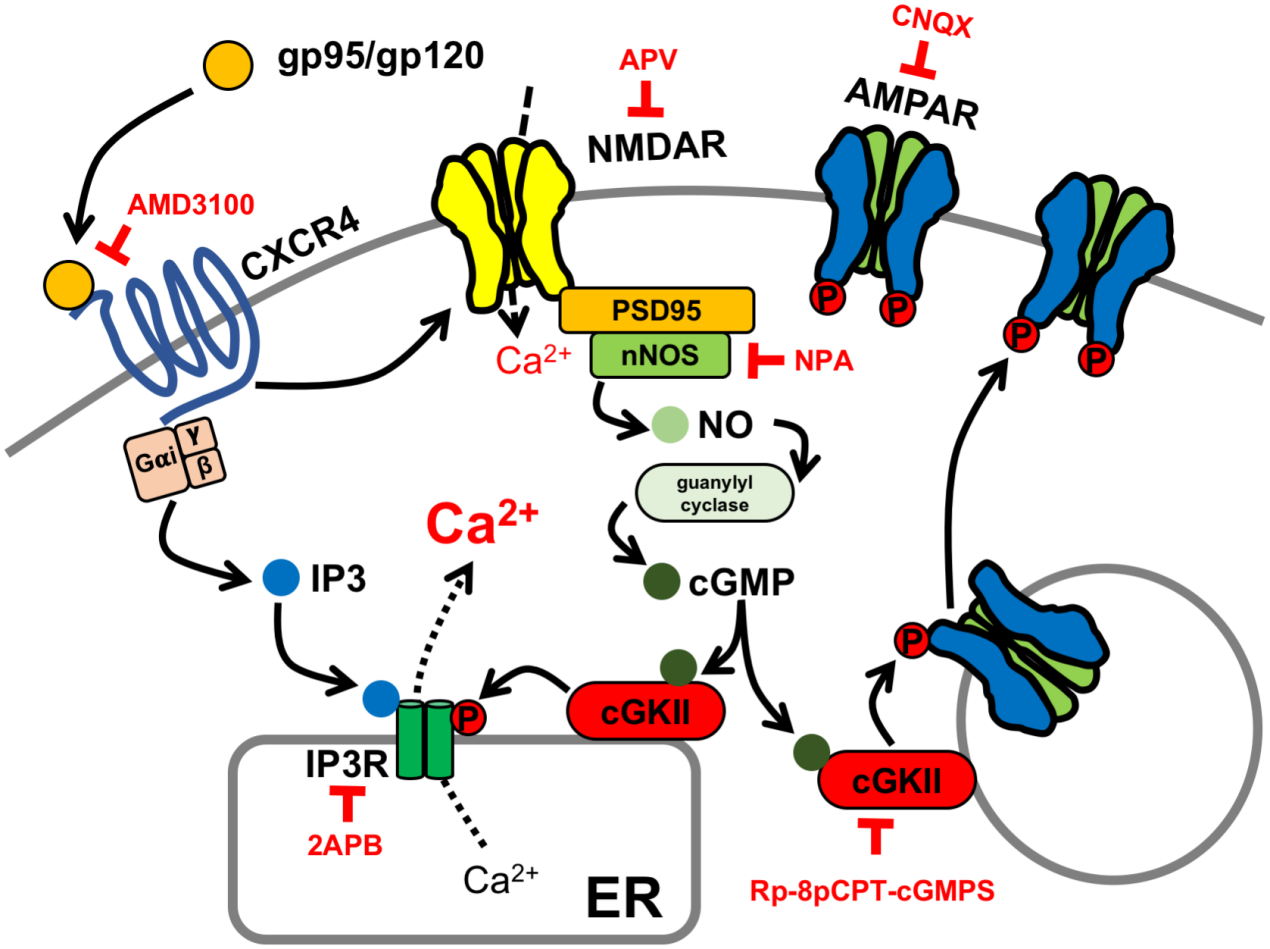
Model for gp95/120-induced activity-dependent synaptic hyperexcitation. Both FIV gp95 and HIV gp120 stimulate CXCR4 and NMDARs, inducing activity-dependent synaptic dysfunction via cGKII activation. The gp95/120 stimulation of NMDARs activates nNOS, production of NO, leading to activation of soluble guanylyl cyclase and the production of cGMP, which in turn activates cGKII. Both the production of IP3 by the gp95/120 stimulation of CXCR4 and cGKII-induced phosphorylation of IP3Rs enhance ER Ca^2+^ release, contributing to Ca^2+^ hyperactivity. In addition, gp95-induced cGKII activation increases GluA1 phosphorylation, promoting elevation of surface AMPARs, which leads to the elevation of synaptic excitation. Therefore, the gp120/gp95-induced stimulation of cGKII is critical for synaptic hyperexcitation in HAND pathophysiology. AMD3100, bicyclam derivative plerixafor hydrochloride; AMPAR, AMPA receptor; cGKII, cGMP-dependent protein kinase II; cGMP, cyclic GMP; CNQX, 6-Cyano-7-nitroquinoxaline-2,3-dione; ER, endoplasmic reticulum; FIV, feline immunodeficiency virus; HAND, HIV-associated neurocognitive disorder; HIV, human immunodeficiency virus; IP3R, inositol triphosphate receptor; NMDAR, NMDA receptor; nNOS, neuronal nitric oxide synthase; NO, nitric oxide; NPA, *N*^ω^-Propyl-L-arginine hydrochloride; PSD95, postsynaptic density 95.

Although synaptic dysfunction, not neuronal death, is strongly associated with HAND [5], the molecular mechanisms underlying HAND-associated synaptic impairment remain largely unclear [6]. Previous studies document that FIV envelope proteins also elevate neuronal Ca^2+^ and induce cell death in neurons [22,62,63]. However, cellular mechanisms of such FIV envelope protein-induced neurotoxic effects are unknown. We reveal that FIV envelope glycoprotein gp95 binds to CXCR4 on the neuronal plasma membrane and subsequently elevates intracellular Ca^2+^ through mobilizing ER Ca^2+^ via the stimulation of IP3Rs, as well as NMDARs, the same pathway of HIV gp120-induced Ca^2+^ overactivation [18,49,50] (Fig 8). Most notably, our study identifies that gp95-stimulated NMDARs activate the nNOS-cGMP-cGKII pathway, which subsequently phosphorylates IP3Rs and AMPAR subunit GluA1, leading to the elevation of surface GluA1 expression and AMPAR-mediated synaptic activity, a cellular basis of synaptic dysfunction in HAND (Fig 8). Moreover, we show that cGKII activation is required for Ca^2+^ hyperactivity caused by HIV gp120 (Fig 7A and 7B), suggesting that cGKII activation plays crucial roles in synaptic dysfunction in both HIV and FIV models and there is a conserved cellular pathophysiology from mice and cats to humans.

Although treatment of a lower dose of CNQX or DL-APV was unable to inhibit basal Ca^2+^ activity, lower doses in combination completely inhibited Ca^2+^ activity in both control and gp95-treated neurons (S5 Fig), suggesting that inhibition of both receptors induces additive effects on Ca^2+^ activity. Given that there is NMDAR-independent Ca^2+^ influx via L-type voltage-gated Ca^2+^ channels [64], a lower dose of DL-APV alone is unable to block neuronal Ca^2+^ activity completely. In fact, we found that 10-μM nifedipine, an antagonist of L-type voltage-gated Ca^2+^ channels, was sufficient to abolish GCaMP5 activity in both control and gp95-treated cells (S6B Fig). In addition, NMDARs significantly contribute to signaling at rest in the absence of AMPAR activity [65], although Ca^2+^ permeability through NMDARs at negative membrane potentials is restricted because of their blockade by extracellular Mg^2+^ ions [66,67]. Taken together, although AMPAR-mediated dendritic depolarization is required for removal of Mg^2+^ ions for NMDAR activity, both receptors can also contribute to neuronal Ca^2+^ activity in parallel.

We found that both amplitude and frequency of Ca^2+^ activity and mEPSCs are significantly elevated in gp95-treated neurons (Figs 1B and 4E). Although the current study identifies cellular mechanisms of gp95-induced postsynaptic changes, it is possible that there can be gp95-induced presynaptic processes that induce synaptic hyperexcitation. Notably, neuroinflammatory processes mediated by activated microglia have been strongly implicated in a number of neurodegenerative diseases, including HAND [68]. Similar to neuronal mechanisms, HIV gp120 interacts with microglial CXCR4 and stimulates cGMP-dependent kinase, leading to microglial activation during neurodegenerative inflammation [69]. Importantly, gp120 elevates synaptic receptor activity by enhancing the release of pro-inflammatory cytokines from activated microglia [70,71]. Among those cytokines, tumor necrosis factor alpha (TNFα) induces a rapid increase in mEPSC amplitude and frequency [72–74], as seen in gp95-treated neurons (Fig 4E). Although we used Neurobasal Medium designed for significantly less proliferation of glia [75], we were unable to completely remove microglia in our culture. This thus suggests that microglial activation by gp120 and gp95 can promote TNFα release, resulting in elevation of mEPSC frequency and amplitude.

Both HIV gp120 and FIV gp95 interact with CXCR4 to produce IP3, which can induce IP3R-mediated Ca^2+^ efflux from the ER (Fig 2). However, we found that TTX completely abolished Ca^2+^ activity (S6 Fig). This suggests that IP3 production through the interaction between gp120/gp95 and CXCR4 is not sufficient to increase neuronal Ca^2+^ activity in the absence of neuronal activity. Furthermore, gp95-induced stimulation of AMPARs and NMDARs is insufficient to elevate Ca^2+^ activity in neuronal cell bodies in the absence of IP3R-mediated ER Ca^2+^ release (Fig 8), as indicated by decreased Ca^2+^ activity via the inhibition of ER Ca^2+^ channels (Fig 2). In our model, we thus propose that neuronal activity–driven stimulation of extracellular Ca^2+^ influx through L-type voltage-gated Ca^2+^ channels and NMDARs and Ca^2+^ efflux from the ER coordinate and contribute to somatic Ca^2+^ activity (Fig 8). Further work is necessary to differentiate the roles of these Ca^2+^ channels on HIV-induced neuronal hyperexcitability.

The chemokine receptors CCR5 and CXCR4 are co-receptors together with CD4 for HIV entry into target cells [17]. Macrophage-tropic HIV viruses use CCR5 as a co-receptor [76–80], whereas T-cell line–tropic viruses use CXCR4 [81,82]. Given that most of the HIV-infected cells in the brain are macrophages and microglia, it is thought that CCR5 strains of HIV are the predominant viral species in the brain [83,84]. However, once HIV infection is established, dual tropic and CXCR4-preferring viruses slowly evolve from macrophage-tropic HIV viruses as an indication of progression to AIDS and HIV-associated dementia [17, 85–88]. Moreover, CXCR4- and dual-tropic strains of HIV have been isolated from the brains of infected individuals [86]. Therefore, CXCR4-tropic strains of HIV also play critical roles in the pathogenesis of HAND. Several studies have shown that the HIV gp120 binding to both CCR5 and CXCR4, even without CD4, contributes to neuronal injury and death both in vitro and in vivo [29–31, 89–93]. Interestingly, a CCR5 antagonist prevents gp120 neurotoxicity [94, 95], and natural CCR5 ligands confer protection upon neurons against gp120 toxicity [61]. Conversely, HIV-induced apoptosis can be prevented by AMD3100, a CXCR4 antagonist, both in vitro and in vivo [96–98]. This suggests that CXCR4-mediated signaling can trigger HIV-induced neurotoxicity while CCR5 either protects or disrupts the CNS, depending on the context, ligand characteristics, and resultant signaling pathway. Surprisingly, CCR5-tropic gp120 (JRFL) also requires cGKII activation to induce Ca^2+^ hyperexcitation (Fig 7B). It has been shown that chemokines and their receptors coordinate the signaling at the immunological synapses. In fact, during T-cell activation, CXCR4 and CCR5 chemokine receptors are recruited into the immunological synapse by antigen-presenting cell-derived chemokines [99]. In addition, the co-stimulatory properties of CCR5 and CXCR4 depend on their ability to form heterodimers [100]. Thus, gp120 (JRFL)-induced stimulation to CCR5 can interact with CXCR4, resulting in cGKII activation. Notably, the natural ligand of CXCR4, SDF-1, is also sufficient to induce cGKII-dependent Ca^2+^ overactivation (Fig 7C). Taken together, CCR5–CXCR4 stimulation is sufficient to induce hyperexcitation in neurons. While both CXCR4 and CCR5 are important in the neuropathogenesis of HIV, it is clear that further study of the downstream pathways of CCR5 and CXCR4 activation in neurons will widen the understanding of HIV-induced neuronal toxicity. Given that FIV also targets primary CD4 T cells but uses CD134 instead of CD4 as a primary receptor and uses its sole co-receptor CXCR4 for efficient infection of target cells, similarly to T cell–tropic strains of HIV [18,19,49], FIV infection of cats is an ideal in vivo model to investigate CXCR4-mediated neuropathology in chronic HIV infection.

Our work extends beyond understanding of molecular mechanisms underlying HIV-induced neuronal dysfunction. One of the challenges that the HAND research community has faced in the treatment of this disorder is the lack of a viable target [1]. By identifying cGKII as the downstream effector of the gp95/120-induced synaptic hyperexcitation, our study completes the pathway and identifies cGKII as a new therapeutic target for limiting gp95/120-induced synaptic dysfunction. Moreover, we reveal that CCR5-tropic gp120-induced Ca^2+^ overactivation is also dependent on cGKII (Fig 7B). This thus suggests that cGKII activation is important for CCR5 and CXCR4-dependent neuropathology in HAND. Inhibition of cGKII may be superior as a therapeutic target to other forms of ER Ca^2+^ release control, as its inhibition will limit the NMDAR-induced and IP3R phosphorylation–dependent Ca^2+^ increase specifically, which are likely to be elevated under hyperexcitable conditions, while leaving basal functions unchanged. Thus, use of cGKII inhibition as a means for neuroprotection in individuals infected with HIV is a novel and innovative approach to this therapeutically challenging disease pathway.

## Materials and methods

### Ethics statement

Colorado State University’s Institutional Animal Care and Use Committee reviewed and approved the animal care and protocol (16-6779A).

### Mouse and feline neuron culture

Mouse hippocampal and cortical neuron cultures were prepared as described previously [43,46,47]. Neurons were isolated from embryonic day 17–18 or postnatal day 0.5 C57Bl6 or cGKII KO mouse embryonic brain tissues. For feline hippocampal neurons, embryos were obtained by cesarean section at approximately 35–40 days gestation from specific pathogen-free cats. Hippocampi were isolated from embryos and digested with 10 U/mL papain (Worthington Biochemical Corp., NJ). Mouse cortical neurons were plated on polylysine-coated 15-cm dishes (25 million cells per dish) and 6-well dishes (500,000 cells per well) for biochemical experiments. Mouse and feline hippocampal cells were prepared in glass-bottom dishes (500,000 cells in the glass bottom) for Ca^2+^ imaging. Mouse hippocampal neurons were also plated on 12-mm coverslips for electrophysiology (200,000 cells per coverslip). Cells were grown in Neurobasal Medium with B27 and 0.5 mM Glutamax and penicillin/streptomycin (Life Technologies).

### Reagents

Expression, amplification, and purification of FIV envelope glycoprotein gp95 and capsid p26 recombinant proteins were performed using previously described methods [50,101]. Briefly, gp95 was purified from Chinese hamster ovary (CHO) cells, and the human Fc tag, in frame with either protein, served as a means to purify the proteins using *Staphylococcus* Protein A-Sepharose [102,103], or was isolated following transfection of HEK 293S cells with an expression vector. Gag antigen was expressed in *Escherichia coli* and purified using a GST-tag [101]. HIV CXCR4-tropic gp120 (IIIB) was obtained through the NIH AIDS Reagent Program, Division of AIDS, NIAID, NIH: HIV-1 IIIB gp120 Recombinant Protein from ImmunoDX, LLC. HIV CCR5-tropic gp120 (JRFL) was obtained from Dr. C.A.L. Kassuya at the Federal University of Grande Dourados, Brazil [104]. The following inhibitors were used in this study: 200-nM AMD3100 (Tocris), 25-μM 2APB (Abcam), 10-μM Dantrolene (Abcam), 25-μM or 50-μM DL-APV (Abcam), 2-μM NPA (Tocris), 1-μM or 10-μM Rp8-Br-PET-cGMPS (Tocris) for cultured mouse or feline neurons, respectively, 10-μM or 30-μM CNQX (Abcam), 1-μM TTX (Tocris), 10-μM nifedipine (Abcam), and 20-nM human SDF-1 (Abcam).

### GCaMP5 Ca^2+^ imaging

GCaMP5 Ca^2+^ imaging was carried out by a modification of the previously reported method [43,46,47]. DIV4 Neurons were transfected with pCMV-GCaMP5 (a gift from Douglas Kin and Loren Looger, Addgene plasmid #31788) [105] by using Lipofectamine 2000 (Life Technologies) according to the manufacturer’s protocol. The transfection efficiency is about 5%, and obvious cellular toxicity has not been observed. Neurons were grown in Neurobasal Medium without phenol red supplemented with B27 and 0.5-mM Glutamax and penicillin/streptomycin (Life Technologies) for 8–12 days after transfection and during the imaging. Glass-bottom dishes were mounted on a temperature-controlled stage on Olympus IX73 and maintained at 37 °C and 5% CO_2_ using a Tokai Hit heating stage and digital temperature and humidity controller. The imaging was captured for periods of 50 milliseconds using a 60× oil-immersion objective. A total of 100 images was obtained with 1-second interval, and Ca^2+^ activity in the cell body (excluding dendrites) was analyzed using the Olympus CellSens software. F_min_ was determined as the minimum fluorescence value during the imaging. Total Ca^2+^ activity was obtained by combining 100 values of ΔF/F_min_ = (F_t_ − F_min_)/F_min_ in each image, and values of ΔF/F_min_ < 0.2 were rejected due to bleaching. Twenty to thirty neurons were used for imaging in one experiment, and one individual neuron was assayed in one imaging.

### Synaptosome purification, surface biotinylation, and immunoblots

Synaptosomal fractions from DIV14 primary cortical neurons were prepared as described previously [43,46,47]. Surface biotinylation was performed according to the previous studies [43,46,47]. For IP3R phosphorylation, whole cell lysates were collected as described previously [43]. Equal amounts of protein were loaded on 10% SDS-PAGE gel and transferred to nitrocellulose membranes. Membranes were blotted with GluA1 (Millipore, 1:2,000), GluA2 (Abcam, 1:2,000), pGluA1(S845) (Millipore, 1:1,000), pIP3R(S1756) (Cell signaling, 1:1,000), tubulin (Sigma, 1:5,000), PSD95 (Neuromab, 1:2,000), synaptophysin (Sigma, 1:5,000), NR1 (Millipore, 1:1,000), and actin (Abcam, 1:2,000) antibodies and developed with ECL (Thermo Fisher Scientific). Synaptosomes were isolated from at least three independent cultures, and immunoblots were at least duplicated for quantitative analysis.

### Electrophysiology

To record mEPSCs, aCSF contained bicuculline (20 μM), TTX (1 μM), and glycine (1 μM). The recording chamber contained aCSF with a composition of (in mM) 119 NaCl, 5 KCl, 2.5 CaCl_2_, 1.5 MgCl_2_, 30 glucose, and 20 HEPES and was kept at a constant temperature of 31.0 °C. Patch pipettes were filled with (in mM) 120 KGlu, 20 KCl, 2 MgCl_2_, 10 HEPES, 2 MgATP, 200-μM GTP, and 12.5 mg sucrose and were pH’d with KOH to 7.4. Cells were voltage clamped at −70 mV and input resistance and series resistance were monitored throughout experiments. mEPSCs were amplified and recorded using pClamp10.3. Mini Analysis Program Demo (Synaptosoft, GA) was used to measure the peak mEPSC amplitude and decay time. CellSens software (Olympus) was used to visualize cells. Patching pipettes were pulled from borosilicate capillary tubing (Sutter Instruments, CA) and the electrode resistance was typically 4–6 mOhms.

### Statistics

Statistical comparisons were analyzed with the GraphPad Prism6 software. Unpaired two-tailed Student *t* tests were used in single comparisons. For multiple comparisons, we used one-way ANOVA followed by Fisher’s Least Significant Difference (LSD) test to determine statistical significance. Results were represented as mean ± SEM, and *p* < 0.05 was considered statistically significant.

## Supporting information

S1 Fig GCaMP5 imaging and analysis. An example of time-lapse images and their responses. A scale bar indicates 10 μm.
(TIF)

S2 Fig NMDAR-dependent gp95-induced Ca^2+^ hyperactivity. Representative traces of GCaMP5 fluorescence intensity and a summary graph of the normalized average of total Ca^2+^ activity in each condition showing that a higher dose of DL-APV completely blocks Ca^2+^ activity in both control and gp95-treated neurons, while a lower dose of DL-APV selectively inhibits the gp95 effects (*n* = number of neurons, ****p < 0.0001, one-way ANOVA, uncorrected Fischer’s LSD, F (5,304) = 9.238). A scale bar indicates 20 seconds. LSD, Least Significant Difference; NMDAR, NMDA receptor.
(TIF)

S3 Fig Control for synaptosomal fractions. The quality of synaptosomes used in Fig 4A and 4B has been monitored by immunoblots of synaptic proteins in sequential fractions showing that synaptic proteins such as GluA2, PSD95, and synaptophysin are highly enriched in the synaptosome fractions. PSD95, postsynaptic density 95.
(TIF)

S4 Fig Gp95 is unable to alter surface expression of GluA2 and NR1. Representative immunoblots and quantitative analysis of surface biotinylation in each condition showing that gp95 has no effect on surface expression of **(A)** GluA2 (*n* = 12 experiments) and **(B)** NR1 (*n* = 5 experiments, CTRL). Actin is used as an intracellular negative control and absent in the biotinylated samples.
(TIF)

S5 Fig AMPAR-dependent gp95-induced Ca^2+^ hyperactivity. Representative traces of GCaMP5 fluorescence intensity and a summary graph of the normalized average of total Ca^2+^ activity in each condition showing that a higher dose of CNQX completely blocks Ca^2+^ activity in both control and gp95-treated neurons, while a lower dose of CNQX selectively inhibits the gp95 effects. Furthermore, lower doses of DL-APV and CNQX in combination completely inhibited Ca^2+^ activity in both control and gp95-treated neurons, suggesting that inhibition of both receptors induces additive effects on Ca^2+^ activity (*n* = number of neurons, ****p < 0.0001, one-way ANOVA, uncorrected Fischer’s LSD, F (7,179) = 5.933). A scale bar indicates 20 seconds. AMPAR, AMPA receptor; CNQX, 6-Cyano-7-nitroquinoxaline-2,3-dione; LSD, Least Significant Difference.
(TIF)

S6 Fig GCaMP5 activity is mediated by neuronal activity and L-type Ca^2+^ channels. Representative traces of GCaMP5 fluorescence intensity and a summary graph of the normalized average of total Ca^2+^ activity in each condition showing that inhibition of **(A)** neuronal activity by TTX and **(B)** L-type Ca^2+^ channels abolish GCaMP5 activity in both control and gp95-treated neurons (*n* = number of neurons, ****p < 0.0001, one-way ANOVA, uncorrected Fischer’s LSD, **(A)** F (3,220) = 25.61 and **(B)** F (3,206) = 17.17). A scale bar indicates 20 seconds. LSD, Least Significant Difference; TTX, tetrodotoxin.
(TIF)

S1 Data Contains raw numerical values that underlie the summary data displayed in the following figure panels: Figs 1A–1C, 2, 3A–3D, 4A–4E, 5, 6, 7A–7C, S2, S4A, S4B, S5, S6A and S6B. (XLSX)

## Abbreviations

2APB: 2-aminoethoxydiphenyl borate
a.u.: arbitrary unit
AIDS: acquired immune deficiency syndrome
AMD3100: bicyclam derivative plerixafor hydrochloride
AMPAR: α-amino-3-hydroxy-5-methyl-4-isoxazolepropionic acid receptor
ANI: asymptomatic neurocognitive impairment
cART: combination antiretroviral therapy
CCR5: C-C chemokine receptor type 5
CD: cluster of differentiation
cGKII: cGMP-dependent protein kinase II
cGMP: cyclic guanosine monophosphate
CHO: Chinese hamster ovary
CNQX: 6-Cyano-7-nitroquinoxaline-2,3-dione
CNS: central nervous system
CP-AMPAR: Ca^2+^-permeable AMPAR
CXCR4: C-X-C chemokine receptor type 4
DIV: day in vitro
DL-APV: DL-2-Amino-5-phosphonopentanoic acid
ER: endoplasmic reticulum
FIV: feline immunodeficiency virus
HAND: HIV-associated neurocognitive disorder
HIV: human immunodeficiency virus
IP3: inositol triphosphate
IP3R: inositol triphosphate receptor
KO: knockout
LSD: Least Significant Difference
LTP: long-term potentiation
mEPSC: miniature excitatory postsynaptic current
MND: mild neurological disorder
NASPM: 1-naphthyl acetyl spermine
NMDAR: N-methyl-D-aspartate receptor
nNOS: neuronal nitric oxide synthase
NO: nitric oxide
NPA: *N*^ω^-Propyl-L-arginine hydrochloride
PSD95: postsynaptic density 95
RyR: Ryanodine receptor
SDF-1: stromal cell-derived factor-1
SIV: simian immunodeficiency virus
TNFα: tumor necrosis factor alpha
TTX: tetrodotoxin
