# Supplementary figures and images for: HIV induces synaptic hyperexcitation via cGMP-dependent protein kinase II activation in the FIV infection model
Source: PLoS Biol. 2018 Jul 27;16(7):e2005315. doi: 10.1371/journal.pbio.2005315 (PMC6082575; doi:10.1371/journal.pbio.2005315)

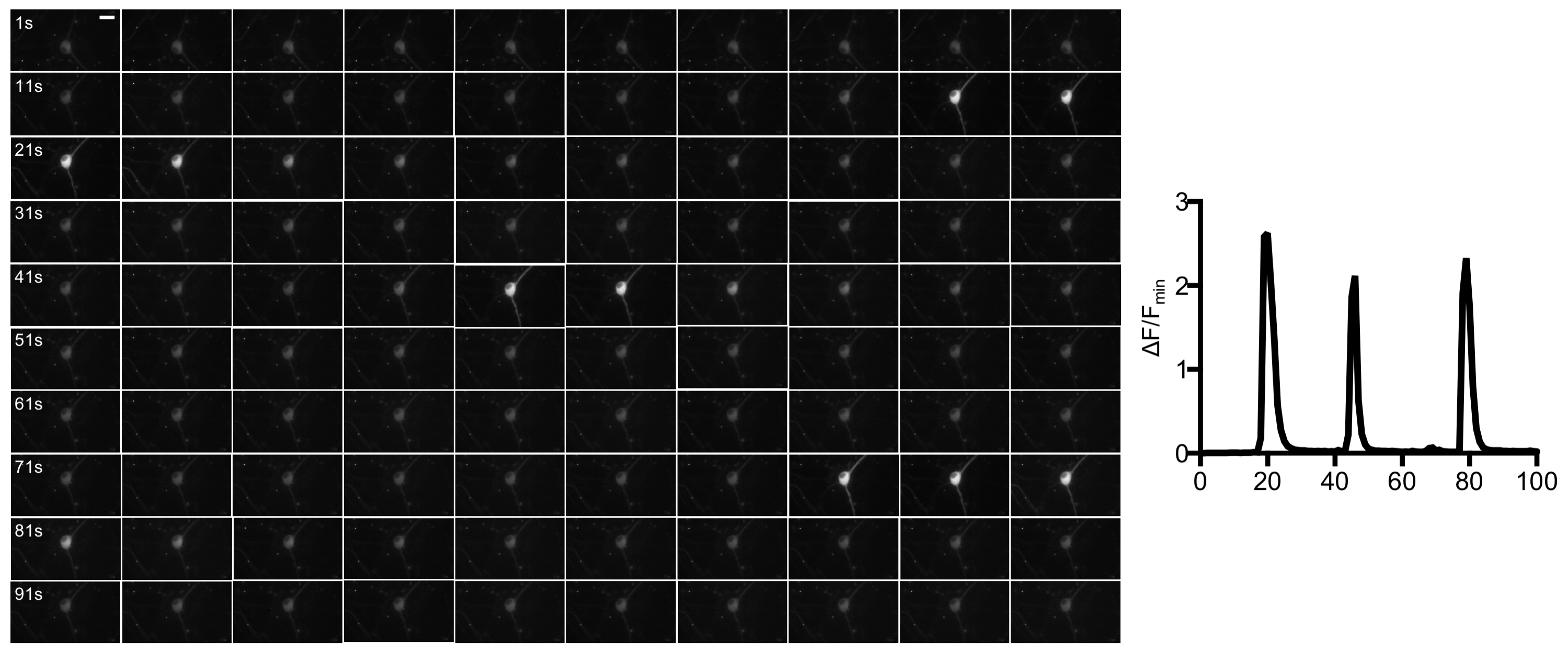

Supplement: S1 Fig — An example of time-lapse images and their responses. A scale bar indicates 10 μm. (TIF) [file pbio.2005315.s001.tif]

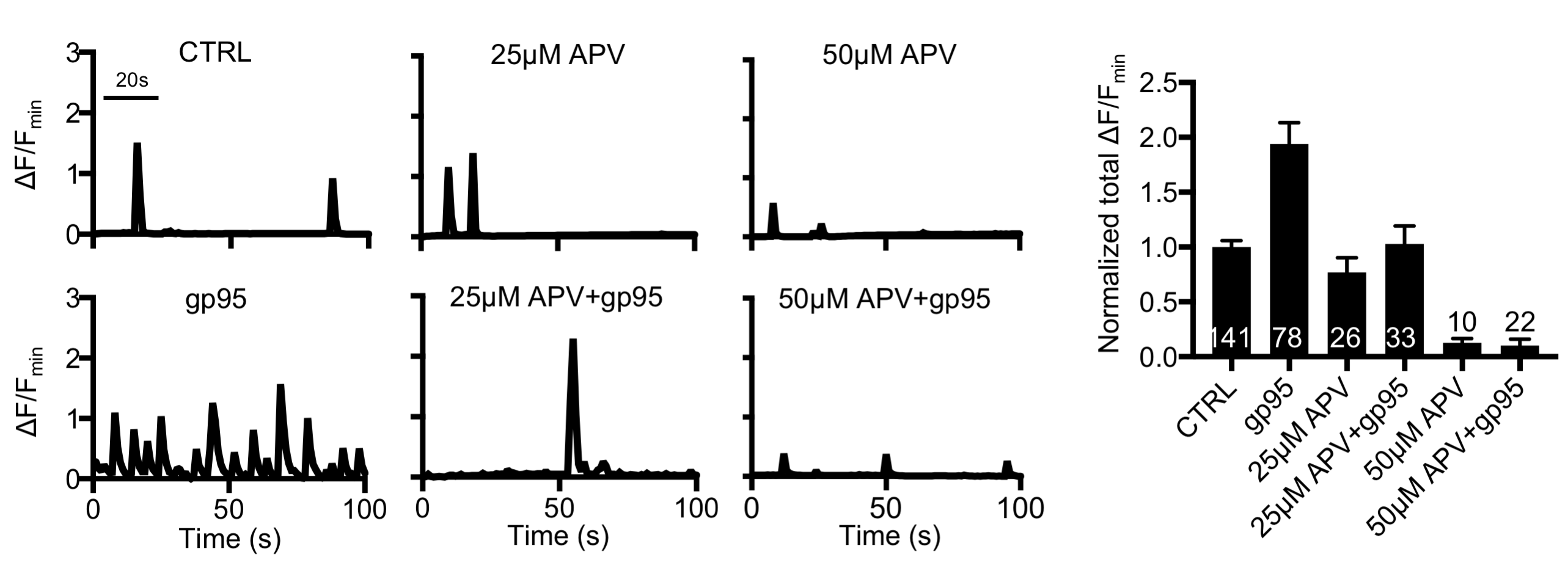

Supplement: S2 Fig — Representative traces of GCaMP5 fluorescence intensity and a summary graph of the normalized average of total Ca2+ activity in each condition showing that a higher dose of DL-APV completely blocks Ca2+ activity in both control and gp95-treated neurons, while a lower dose of DL-APV selectively inhibits the gp95 effects (n = number of neurons, ****p < 0.0001, one-way ANOVA, uncorrected Fischer’s LSD, F (5,304) = 9.238). A scale bar indicates 20 seconds. LSD, Least Significant Difference; NMDAR, NMDA receptor. (TIF) [file pbio.2005315.s002.tif]

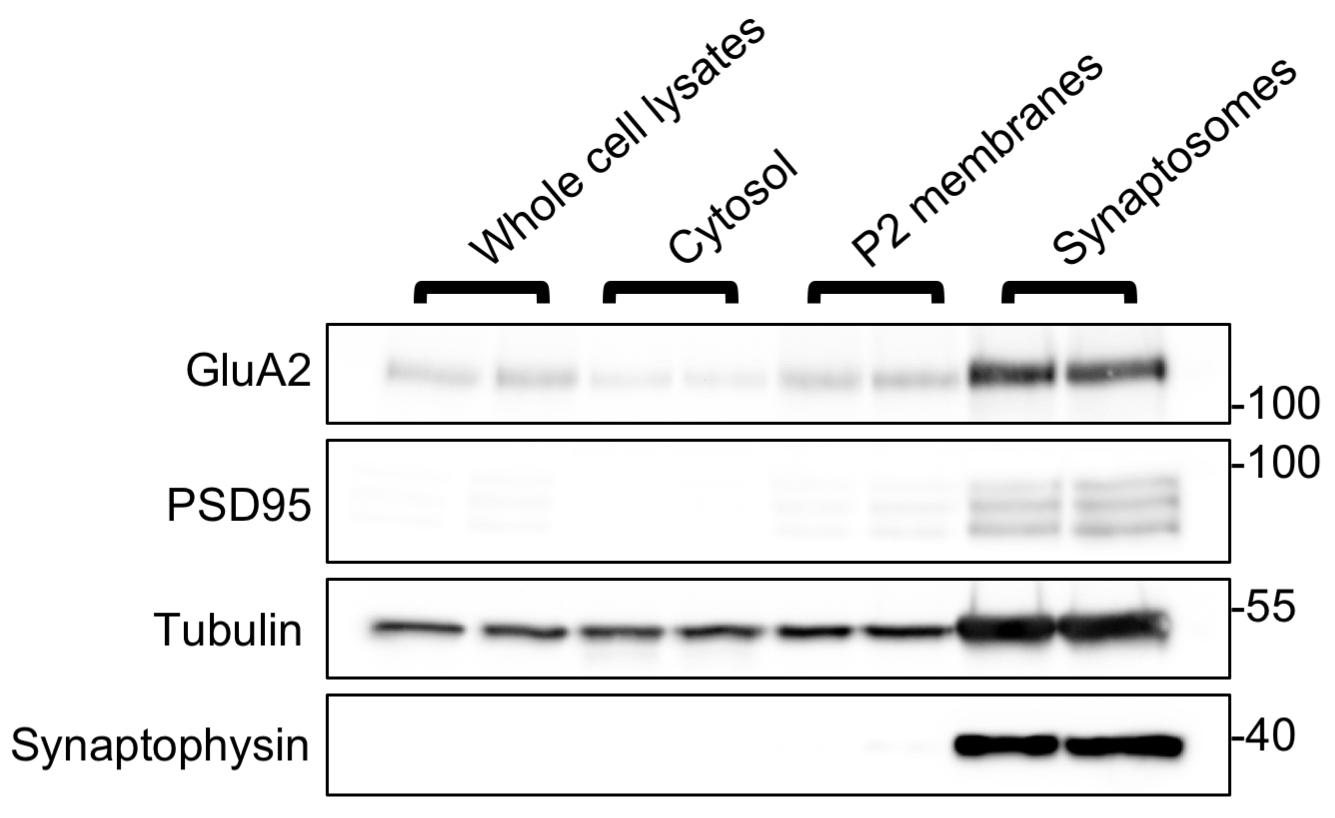

Supplement: S3 Fig — The quality of synaptosomes used in Fig 4A and 4B has been monitored by immunoblots of synaptic proteins in sequential fractions showing that synaptic proteins such as GluA2, PSD95, and synaptophysin are highly enriched in the synaptosome fractions. PSD95, postsynaptic density 95. (TIF) [file pbio.2005315.s003.tif]

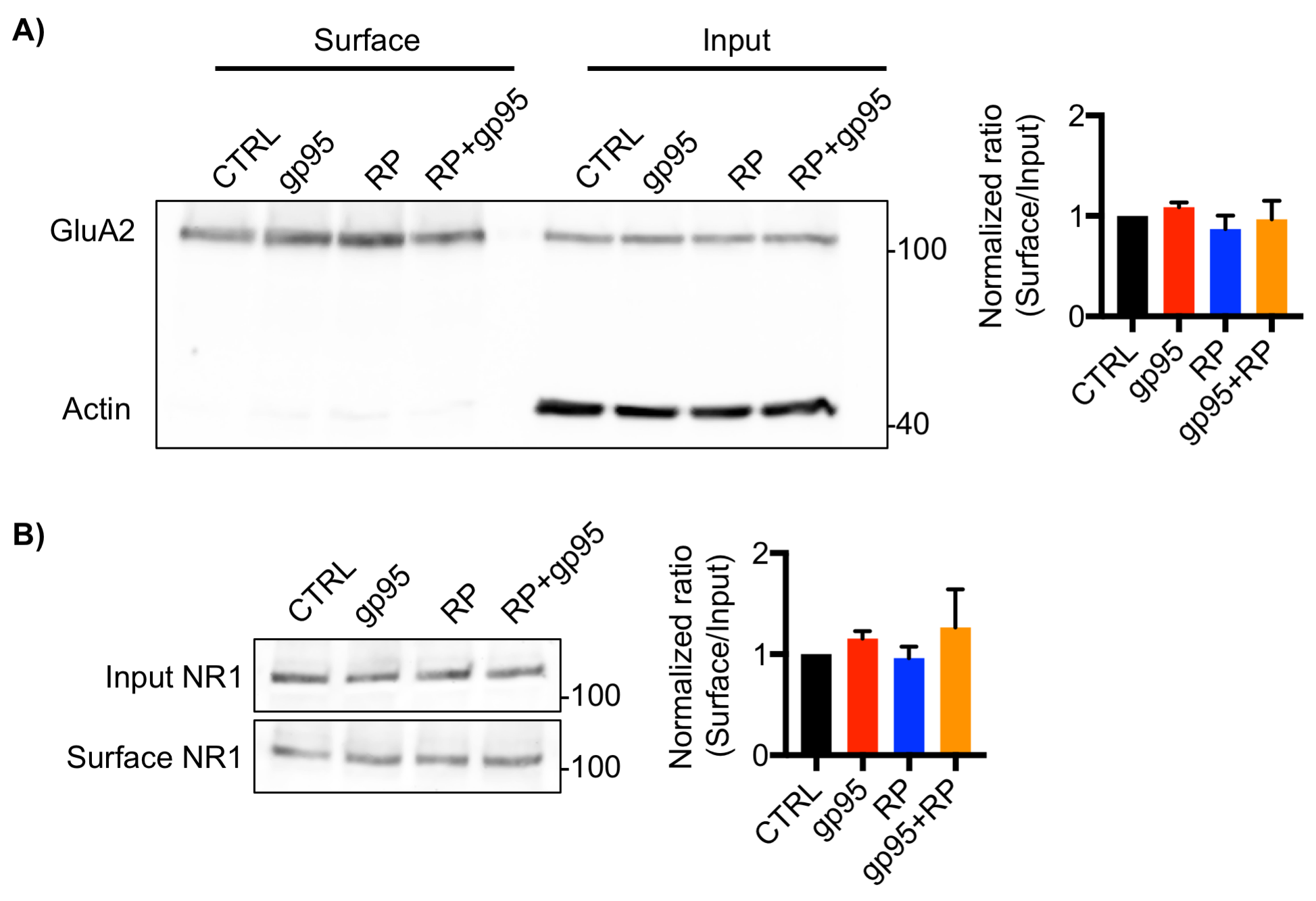

Supplement: S4 Fig — Representative immunoblots and quantitative analysis of surface biotinylation in each condition showing that gp95 has no effect on surface expression of (A) GluA2 (n = 12 experiments) and (B) NR1 (n = 5 experiments, CTRL). Actin is used as an intracellular negative control and absent in the biotinylated samples. (TIF) [file pbio.2005315.s004.tif]

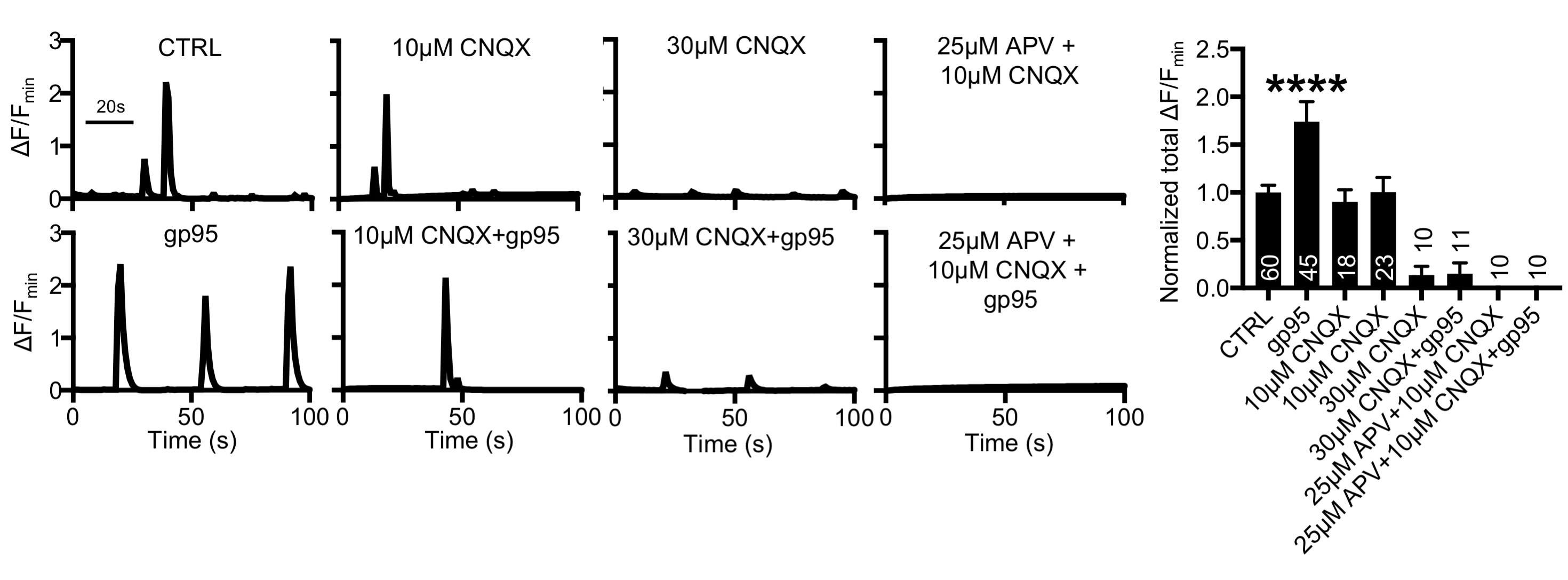

Supplement: S5 Fig — Representative traces of GCaMP5 fluorescence intensity and a summary graph of the normalized average of total Ca2+ activity in each condition showing that a higher dose of CNQX completely blocks Ca2+ activity in both control and gp95-treated neurons, while a lower dose of CNQX selectively inhibits the gp95 effects. Furthermore, lower doses of DL-APV and CNQX in combination completely inhibited Ca2+ activity in both control and gp95-treated neurons, suggesting that inhibition of both receptors induces additive effects on Ca2+ activity (n = number of neurons, ****p < 0.0001, one-way ANOVA, uncorrected Fischer’s LSD, F (7,179) = 5.933). A scale bar indicates 20 seconds. AMPAR, AMPA receptor; CNQX, 6-Cyano-7-nitroquinoxaline-2,3-dione; LSD, Least Significant Difference. (TIF) [file pbio.2005315.s005.tif]

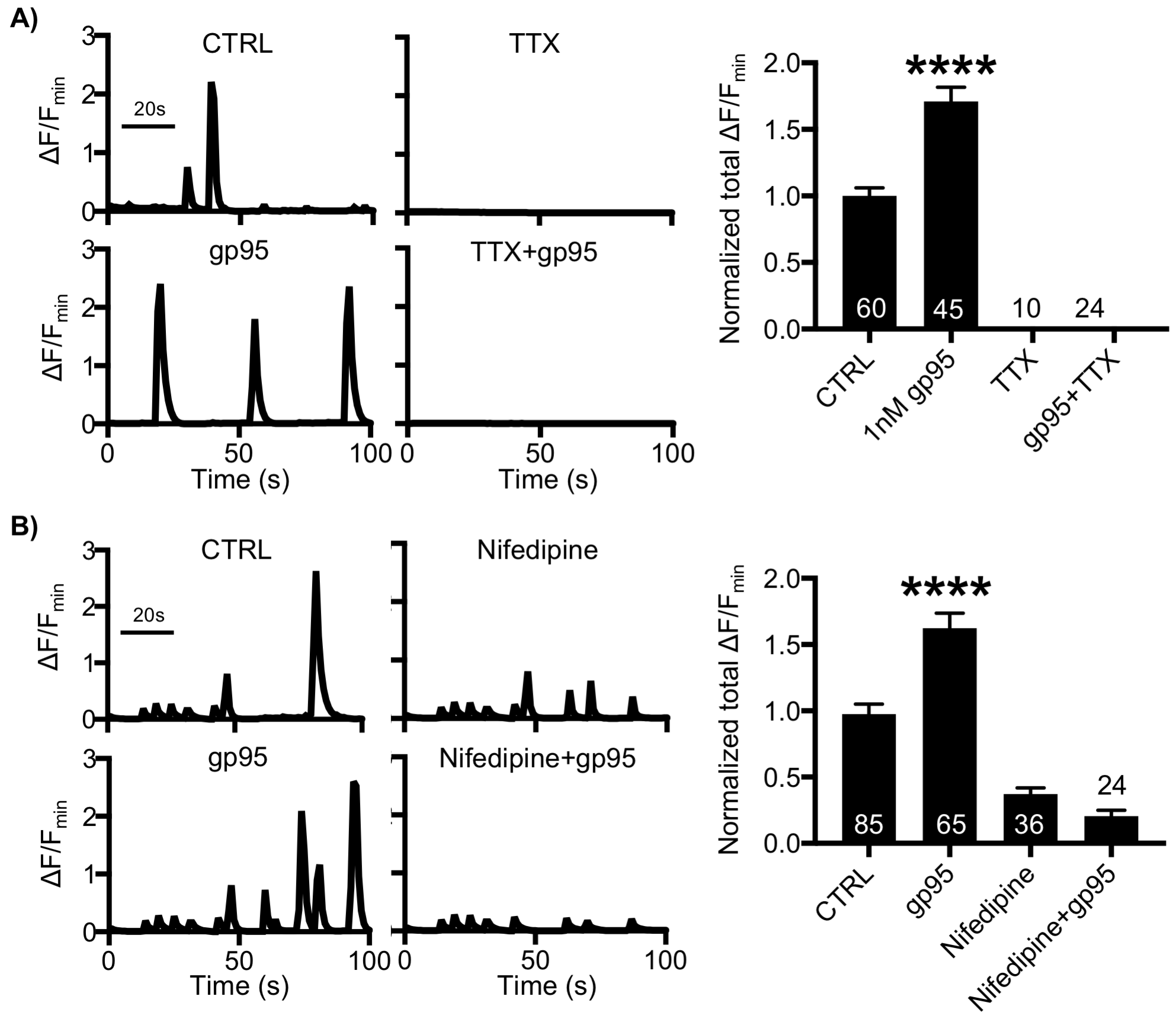

Supplement: S6 Fig — Representative traces of GCaMP5 fluorescence intensity and a summary graph of the normalized average of total Ca2+ activity in each condition showing that inhibition of (A) neuronal activity by TTX and (B) L-type Ca2+ channels abolish GCaMP5 activity in both control and gp95-treated neurons (n = number of neurons, ****p < 0.0001, one-way ANOVA, uncorrected Fischer’s LSD, (A) F (3,220) = 25.61 and (B) F (3,206) = 17.17). A scale bar indicates 20 seconds. LSD, Least Significant Difference; TTX, tetrodotoxin. (TIF) [file pbio.2005315.s006.tif]
